# Impact of collection conditions on the metabolite content of human urine samples as analyzed by liquid chromatography coupled to mass spectrometry and nuclear magnetic resonance spectroscopy

**DOI:** 10.1007/s11306-014-0764-5

**Published:** 2014-12-23

**Authors:** Aurélie Roux, Etienne A. Thévenot, François Seguin, Marie-Françoise Olivier, Christophe Junot

**Affiliations:** 1 Laboratoire d’Etude du Métabolisme des Médicaments, DSV/iBiTec-S/SPI, MetaboHUB Paris, CEA - Centre d’Etude de Saclay, 91191 Gif-Sur-Yvette, France; 2 CEA, LIST, Laboratory for Data Analysis and Smart Systems, MetaboHUB Paris, 91191 Gif-Sur-Yvette, France; 3 INSERM U1082, Université de Poitiers, Hôpital La Milêtrie, Poitiers, France; 4 Laboratoire de Recherche sur la Transcription et la Réparation des Cellules Souches, DSV/IRCM, CEA, Fontenay-Aux-Roses, 92265 France

**Keywords:** Metabolomics, Human urine, Stability, High resolution mass spectrometry

## Abstract

There is a lack of comprehensive studies documenting the impact of sample collection conditions on metabolic composition of human urine. To address this issue, two experiments were performed at a 3-month interval, in which midstream urine samples from healthy individuals were collected, pooled, divided into several aliquots and kept under specific conditions (room temperature, 4 °C, with or without preservative) up to 72 h before storage at −80 °C. Samples were analyzed by high-performance liquid chromatography coupled to high-resolution mass spectrometry and bacterial contamination was monitored by turbidimetry. Multivariate analyses showed that urinary metabolic fingerprints were affected by the presence of preservatives and also by storage at room temperature from 24 to 72 h, whereas no change was observed for urine samples stored at 4 °C over a 72-h period. Investigations were then focused on 280 metabolites previously identified in urine: 19 of them were impacted by the kind of sample collection protocol in both experiments, including 12 metabolites affected by bacterial contamination and 7 exhibiting poor chemical stability. Finally, our results emphasize that the use of preservative prevents bacterial overgrowth, but does not avoid metabolite instability in solution, whereas storage at 4 °C inhibits bacterial overgrowth at least over a 72-h period and slows the chemical degradation process. Consequently, and for further LC/MS analyses, human urine samples should be kept at 4 °C if their collection is performed over 24 h.

**Electronic supplementary material:**

The online version of this article (doi:10.1007/s11306-014-0764-5) contains supplementary material, which is available to authorized users.

## Introduction

Metabolomics deals with the comprehensive analysis of metabolites present in a biological sample by the combined use of analytical methods and statistical analysis. The metabolic fingerprints from tissues or biofluids contain a few hundred to thousands of signals which are related to both genetic and environmental contributions (e.g., lifestyle, gut microbial activity, or exposure to xenobiotics) (Holmes et al. 2008). Metabolomics investigates profile changes in metabolites that reflect variations in metabolism and may provide information regarding pathological state, drug exposure, or biological stress. Metabolome analysis can be done on various tissue or biofluid extracts depending on the kind of study. The use of urine samples is of high interest, especially in clinical chemistry and toxicology, since its collection is non-invasive and concentrations of circulating metabolites are amplified by bladder storage.

Urine is a complex biofluid that contains a variety of different compounds such as amino acids, organic acids, lipids, sugars, hormones, peptides, xenobiotics and end products of metabolism such as glucuronides and sulfoconjugates (Bouatra et al. 2013). The comprehensive analysis of the human urine metabolome has mainly been investigated by NMR, GC–MS and LC/MS (Ryan et al. 2011). Some reference protocols are available for these three technologies (Beckonert et al. 2007; Chan et al. 2011; Want et al. 2013) and some recommendations and standard operating procedures for biobanking have also been published (Barton et al. 2008; Bernini et al. 2011; Dunn et al. 2008).

One important point for metabolomics is the necessity to maximize control of the experimental conditions and their influence on metabolic fingerprints in order to avoid confounding factors that hamper biological interpretations of results. Metabolite stability in biofluids is one of these confounding factors. The stability of proteins and metabolites in human urine has already been investigated under various sampling and storage conditions (Gika et al. 2007; Gika et al. 2008; Lauridsen et al. 2007; Maher et al. 2007; Saude and Sykes 2007; Thongboonkerd and Saetun 2007). Long- and medium-term stabilities seem to be satisfactory over a period of 6 months if the storage temperature is less than or equal to −20 °C (Gika et al. 2008; Lauridsen et al. 2007). Lauridsen et al. observed no change in the ^1^H-NMR fingerprints of urine samples stored at −25 °C for 26 weeks. They recommend storage of urine samples at this temperature or below and showed that addition of preserving agents is not mandatory in these conditions (Lauridsen et al. 2007). Furthermore, two other studies performed using NMR (Maher et al. 2007) and GC/MS (Dunn et al. 2008) aimed at evaluating the impact of sample collection and handling concluded that the effect of storage is minimal compared with inter-individual variations of metabolite concentrations.

Whereas short-term stability of processed samples (i.e., centrifuged, diluted, etc.) before analysis is documented for 48 h at 4 °C (and 24 h at room temperature), at least by metabolomics and proteomics profiling and at the chemometrical level (Gika et al. 2008; Thongboonkerd and Saetun 2007), short- and medium-term stability of crude samples at room temperatures is more problematic, especially in the course of urine collection over several hours (24–48 h) (Maher et al. 2007; Saude and Sykes 2007).

It is during sample collection that bacterial overgrowth is likely to be a matter of concern because it impacts on concentrations of some metabolites (Lauridsen et al. 2007; Maher et al. 2007; Saude and Sykes 2007) and also proteins (Thongboonkerd and Saetun 2007). Indeed, bacterial growth is very rapid and can modify urine composition by consuming and producing metabolites. Although urine of healthy subjects is supposed to be sterile, it can be contaminated through the urethra, which contains commensal bacteria that may be carried by the urine stream (Maskell 2010). This explains why urine collection can be a critical step in metabolomics studies and that sampling conditions must be controlled.

The impact of bacterial overgrowth on the urine proteome has been investigated with pooled normal urine by evaluating different kinds of sample handling protocols (i.e., temperature storage, centrifugation, addition of different concentrations of two preserving agents: sodium azide and boric acid). Bacterial overgrowth was assessed by UV–visible spectrophotometry and Gram staining; and proteins were separated by 2D electrophoresis and detected by MALDI-TOF-MS. After 48-hour storage at room temperature without any preservative, the authors found more than 500 newly presented protein spots, some of which were identified by peptide mass fingerprinting. They concluded that urine samples should be centrifuged and kept at 4 °C, rather than at room temperature during the collection interval. They also recommend the addition of 200 mM boric acid or 10 mM sodium azide (Thongboonkerd and Saetun 2007).

Concerning the urinary metabolome, most studies have been performed using NMR (Lauridsen et al. 2007; Maher et al. 2007; Saude and Sykes 2007). Increased concentrations of glycine, creatine, benzoic, acetic, formic and lactic acids were observed, together with decreased levels of creatinine, and hippuric and citric acids. Regarding published studies dealing with LC/MS-based approaches, they are mainly qualitative and based on multivariate analysis of metabolomics profiles or on visual comparison of spectra (Gika et al. 2007; Gika et al. 2008). This may be due in part to the challenging issue of metabolite identification. Furthermore, the use, in most cases, of individual urine samples hampers the detection of metabolic concentration changes due to stability issues because they may be hidden by the huge inter-individual variability usually observed in this biological medium (Gika et al. 2007; Gika et al. 2008; Lauridsen et al. 2007; Maher et al. 2007; Saude and Sykes 2007).

In this context, the purpose of this study is to provide comprehensive data about the short-term stability of metabolites in human urine during sample collection. To this end, two experiments were designed using pooled urine samples which were subjected to different collection protocols: storage at 4 °C or at room temperature, with or without preservatives over a 72-hour period. Bacterial overgrowth was assessed by turbidimetry, and metabolomics was achieved using ultra-high performance liquid chromatography coupled to a high-resolution mass spectrometry (UHPLC/HRMS) method enabling the detection and identification of a few hundred metabolites in human urine (Roux et al. 2012).

## Materials and methods

### Chemicals

All reagents, chemicals and preservatives were from Sigma (Saint Quentin Fallavier, France). The standard mixtures used for the external calibration of the MS instrument (Calmix-positive, for the positive-ionization mode, consisting of caffeine, L-methionyl-arginyl-phenylalanyl-alanine acetate, and Ultramark 1621; Calmix-negative, for the negative-ionization mode, consisting of same mixture plus sodium dodecyl sulfate and sodium taurocholate) were from Thermo Fisher Scientific (Les Ulis, France). Acetonitrile was from SDS (Peypin, France) and formic acid from Merck (Briare-le-Canal, France). Water was deionized and filtered through a Millipore Milli-Q water purification system.

### Biological material

Urine samples were anonymously collected from laboratory staff. Only the gender of the donor was recorded. Samples were collected on the day of the experiment between 9:30 and 10:30 a.m. (T_0_ = 10:30 a.m.). Two experiments were conducted with different donors over a period of 3 months, always using the same protocol (Table 1). Once urine samples were pooled (to eliminate inter-individual variations), several aliquots were prepared and subjected to different collection conditions (with or without preservatives) and were stored either at room temperature (on the laboratory bench, with recorded temperatures ranging from 19 to 26 °C, as displayed in Table 1) or at 4 °C. Samples were collected at regular time intervals (every 4 or 12 h) between 0 and 72 h and kept at −80 °C until analysis. Samples for UHPLC/HRMS analysis were centrifuged (3,000 rpm for 5 min) and then diluted in deionized water (dilution 1/5:20 μl of urine in 80 μl of water) prior to analysis. For nuclear magnetic resonance (NMR) analysis, urine samples were thawed, and 0.4 mL of urine was added to 0.2 mL of phosphate buffer in D_2_O (0.2 M, pH = 7.00) containing sodium 3-trimethylsilyl[2,2,3,3-2H4]propionate (TSP) (Sigma-Aldrich, USA), and pH adjusted to 7.00 after a 20 min delay. Urine was centrifuged to eliminate cellular fragments and sediments, and 0.5 mL was transferred to a 5 mm NMR tube (New Era, USA).

**Table 1:**
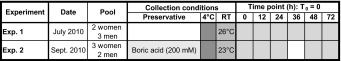
Experimental design. Collection conditions and time points for the two experiments are indicated as colored cells

### Turbidimetry

The turbidity measurement was carried out on a plate reader Multiskan EX from Thermo Fisher Scientific (Les Ulis, France). Absorbances were measured at 620 nm.

### UHPLC/HRMS-based metabolomics

#### Instrumental parameters

Analyses were performed on an Accela liquid chromatographic system (Thermo Fisher Scientific) coupled to an LTQ-Orbitrap Discovery (Thermo Fisher Scientific) fitted with an electrospray source operated in the positive- and negative-ion modes. The software interface was Xcalibur (version 2.1) (Thermo Fisher Scientific). The mass spectrometer was calibrated before each analysis using a calibration solution provided by the manufacturer (external calibration). The UHPLC chromatographic separation was performed on a Hypersil GOLD C18 1.9 μm, 2.1 × 150 mm^2^ column (Thermo Fisher Scientific) equipped with an online prefilter (Interchim, Montluçon, France). The mobile phases were (A) 100 % water and (B) 100 % acetonitrile with 0.1 % formic acid. After an isocratic step of 2 min at 100 % phase A, a linear gradient from 0 to 100 % B was run over the next 11 min with a mobile phase flow of 500 μl/min. After returning to 100 % A at 15.5 min, the column was then allowed to equilibrate for 3.5 min, leading to a total run time of 19 min.

Mass spectra were recorded from 75 to 1,000 Th with an AGC value of 5 × 10^5^. The mass resolution of the analyzer was set to 30,000 (m/Δm, fwhm at 400 Th). The mass spectra were acquired in the reduced profile mode. In the positive-ion mode, the electrospray voltage was set to 5 kV, the capillary voltage to 8 V, and the tube lens offset to 65 V. The sheath and auxiliary gas flows (both nitrogen) were optimized at 35 and 8 arbitrary units (au), respectively, and the drying gas temperature was set to 275 °C. In the negative-ion mode, the electrospray voltage was set to −3.5 kV, and the capillary voltage and tube lens offset were set to −20 and −70 V, respectively; the sheath and auxiliary gas flows (both nitrogen) were 45 and 15 au respectively, and the drying gas temperature was 275 °C.

Biological samples were randomly analyzed and a quality control sample (QC: urine pool) was injected every 10 samples to check the performance of the analytical system in terms of retention times, accurate mass measurements, and signal intensities. All raw data and associated metadata are publically available on the MetaboLights repository (MTBLS148).

#### Data processing

Automatic peak detection and integration were performed using XCMS software package (Smith et al. 2006). Raw files were preliminarily converted to netCDF format with Xconvert (Thermo Scientific, Les Ulis, France). Data were processed using XCMS version 1.14.1 running under R version 2.8.1. The R language was installed on a Dell Eight-core Intel Xeon 3.00 GHz Processor with 16 Go RAM running Linux (Centos 5.2 x86_64). The *matchedFilter* algorithm was used and default values were set for all parameters except for *fwhm*, *step*, *steps*, *mzdiff*, and *mzwid*, which were set respectively at 4, 0.01, 2, 0.01, and 0.01 (for both grouping steps).

#### Intensity drift correction

Intensities of the 4 peak lists (2 experiments × 2 ionization modes) were normalized as follows. First, for each variable, a local quadratic (loess) model representative of intensity variation along passage order was built with the values of the QC sample (Dunn et al. 2011). Intensities were then divided by the model predictions. For the positive peak list of *Exp.* *2*, QC values were not representative, and variable intensities were divided by their median as no obvious drift was observed. Second, peak list intensities were scaled by multiplying each of the four blocks by a common factor equal to the geometric mean of the block raw intensity medians. Note that this last step is only aimed at providing intensities with meaningful values (otherwise relative intensities are close to 1) and does not modify univariate statistics (since nonparametric tests are used) nor multivariate modeling (because of unit-variance scaling). Normalization resulted in a decrease from the median of the coefficient of variation (CV) of the identified variables (see below) within the QC samples in *Exp.* *1* (resp. *Exp.* *2*) from 24 to 5 % (resp. from 14 to 11 %). The few variables that remained correlated with injection order (<5 and 2 % for *Exp.* *1* and *Exp.* *2*, respectively) were discarded.

#### Filtering

Variables with a mean intensity in the QC samples less than two times the mean intensity in blank samples (i.e., phase A: water with 0.1 % formic acid), or with a CV in the QC samples >30 % were filtered out.

#### Statistical analysis

Univariate correlation with time storage duration was tested by using the nonparametric Spearman coefficient of rank correlation (Kvam and Vidakovic 2007) and controlling the false discovery rate (Benjamini and Hochberg 1995) at a 0.01 threshold. All peak intensities of the metabolites showing significant variations were confirmed by manual integration using the QuanBrowser software, in order to avoid any false-positive or false-negative deconvolution that may have been generated with XCMS.

Prior to multivariate analysis, one observation from *Exp.* *2* with outlier distribution quantiles (Alonso et al. 2011) (*p* = 10^−3^ threshold) was discarded. Intensities were log_10_ transformed and variables were mean-centered and unit-variance scaled, prior to principal component analysis (PCA) and partial least-squares (PLS) analysis with two components (with the storage duration as Y response). To assess the predictive performance of each PLS-DA model, cross-validation was used to compute the diagnostic *Q* ^*2*^ value (Wold et al. 2001). The statistical significance of *Q* ^*2*^ was estimated by permutation testing (Szymanska et al. 2012): the *Q* ^*2*^ value was compared to the distribution of 10^3^ *Q* ^*2*^ values corresponding to models built after random permutation of the response labels.

Normalization, univariate and multivariate statistical analyzes were all performed using scripts coded and run in the R language and environment (R Core Team, 2013; version 3.0.2). Venn diagrams were drawn using the “Vennerable” package version 1.0 (Swinton 2009).

#### Metabolite identification

Metabolites were identified by taking into account the metabolomics standard initiative (MSI) criteria (Sumner et al. 2007) and by using a spectral database developed in our laboratory (Roux et al. 2012). UHPLC/HRMS-peaklists generated by XCMS were annotated using an in-house software tool that matches masses (within a 5 ppm range) and retention times of ions of biological datasets with those of reference compounds contained in the spectral database.

### NMR

#### Recording of NMR spectra from human urine

Proton NMR (1H NMR) spectra were acquired with a Bruker Avance-500SB (Bruker, Wissembourg, France) operating at 500.13 MHz and equipped with a 5 mm broadband inverse probe with non-spinning samples and thermostated at 298 K. Spectra were collected into 32 K points with a spectral width of 5,000 Hz (10 ppm). Acquisition of the spectra was performed with a presaturation of the water signal during 2 s after a relaxation delay of 8 s. A total of 128 transients were accumulated for a total acquisition time of 28 min and 24 s. Spectra were phased, baseline corrected, and referenced to TSP manually with the XWIN-NMR software (version 3.5, Bruker, Karlsruhe, Germany).

#### Processing of NMR data

For PCA, spectra were transformed into a table of buckets of size 0.02 ppm with the AMIX software (Version 3.9.7; Bruker Biospin, Germany). PCA were performed with the SIMCA-P software (version 11.0.0.0; Umetrics, Sweden).

The spectral region from 4.50–5.50 ppm was excluded to remove variability due to suppression of the water resonance signal. Each bucket was divided by the total integral of all individual regions to normalize the urine dilution between individual samples. Spectral regions were investigated to identify the discriminating metabolites. Spectra were processed and analyzed with MestReNova sofware (version 6.2.0, Mesrelab, Santiago di compostella, spain). Spectra were obtained after a Fourier transformation without apodization. After phase correction and baseline correction, peak areas were measured by line fitting. Concentrations of molecules were obtained using TSP as quantitative reference.

#### Metabolite identification

Identification of the metabolites was performed using the Human Metabolome Database (Wishart et al. 2007).

## Results and discussion

### Bacterial overgrowth

Initial bacterial contamination of urine occurs during collection. Whereas the bladder urine is sterile in the absence of infection, skin commensal flora (e.g. bacteria of the *Staphylococcus* and *Corynebacterium* genera), but also bacteria of the gastrointestinal tract (*Escherichia*, *Klebsiella*, *Proteus*, *Enterococcus* genera), can be found at the urethral meatus and perineum (Maskell 2010). Consequently, control of bacterial growth during and after urine collection is mandatory.

**Fig. 1:**
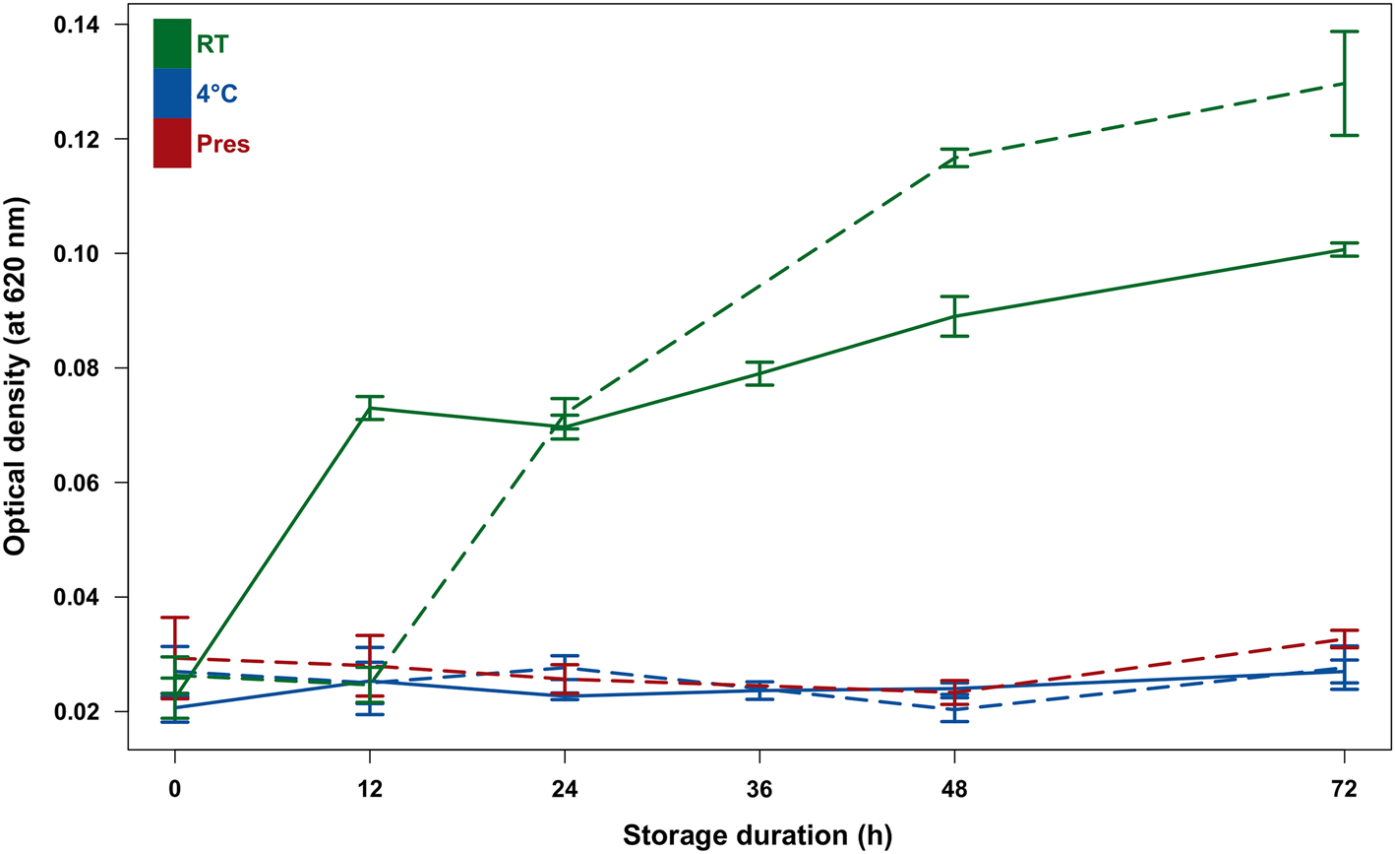
Bacterial growth according to storage conditions. The means and standard deviations of the triplicate optical density measurements are plotted as *solid* (*Exp.* *1*) or *dashed* (*Exp.* *2*) *lines*

Although some variability in bacterial growth was observed at room temperature between the two experiments (Fig. 1), the largest differences occurred within each experiment, according to the experimental factors studied (storage temperature, addition of a preservative). Optical density remained low for all the samples kept at 4 °C. In contrast, bacterial growth increased dramatically at room temperature (i.e., 26 °C and 23 °C for experiment 1, *Exp.* *1*, and experiment 2, *Exp.* *2*, respectively). Such contamination was prevented by adding boric acid preservative to those samples (Fig. 1).

### LC–MS metabolomics analysis of concentration variations induced by urine collection and storage conditions

Preprocessing of the raw LC–MS files (XCMS peak detection and retention time alignment, followed by normalization and QC filtering; see the experimental section for detailed information) resulted in 4 peak Tables (2 experiments × 2 ionization modes) with a number of features ranging from 1,476 to 4,164, which were further analyzed by unsupervised and supervised multivariate modeling.

**Fig. 2:**
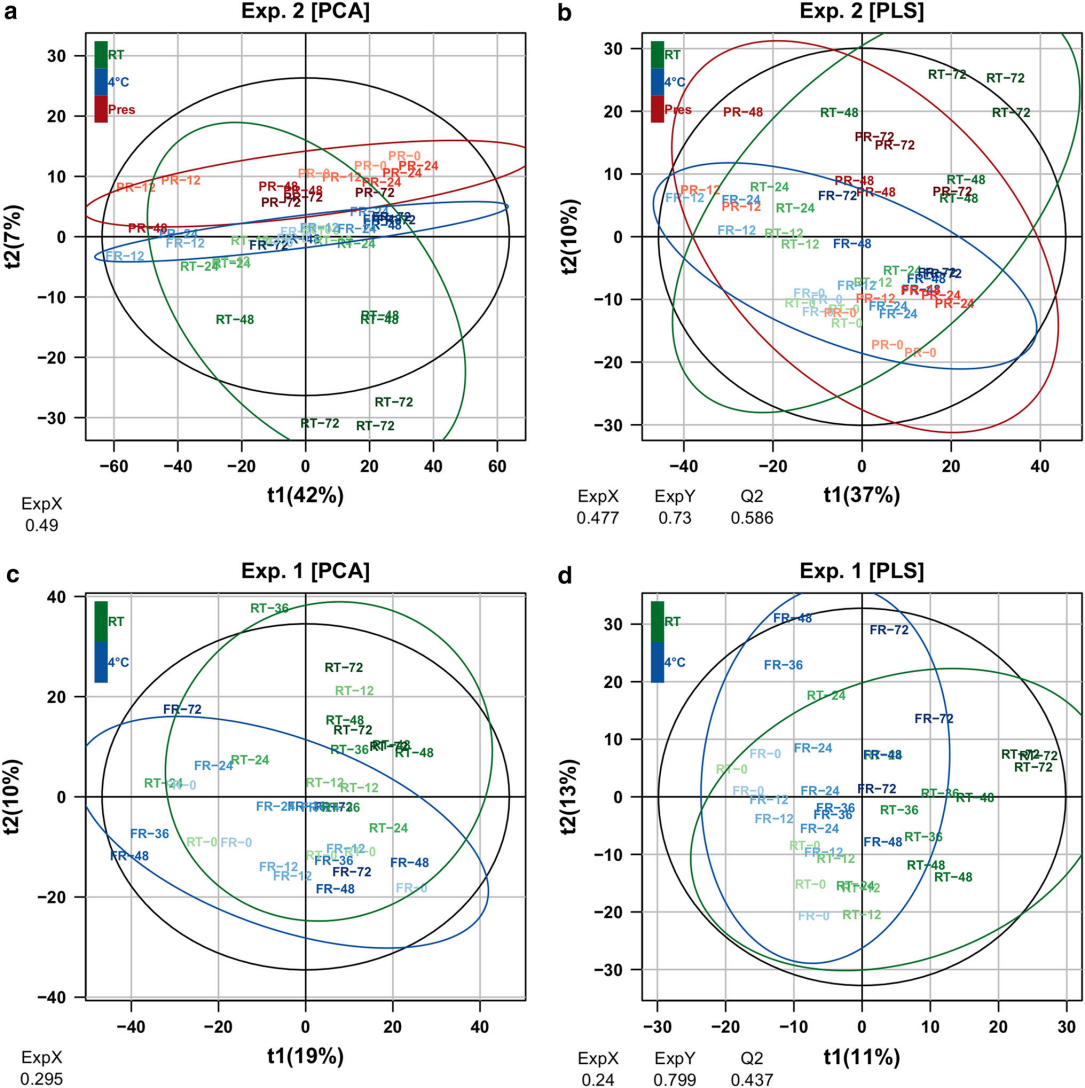
Impact of storage conditions on metabolite concentrations analyzed by multivariate modeling. **a** and **c** Principal component scores of the positive peak tables from *Exp.* *2* (**a**) and *Exp.* *1* (**c**) in the first two dimensions. The individual (in *parentheses*) and cumulative (ExpX) proportions of explained variance are indicated. The 95 %* ellipses* corresponding to a multinormal distribution for each sample condition are superimposed in* color*. **b** and **d** Scores resulting from partial least-squares (PLS) modeling of the storage duration in *Exp.* *2* (**b**) and *Exp.* *1* (**d**). The cumulative proportion of explained variance of the response (ExpY) and the estimation of the model predictive performance by cross-validation (*Q* ^*2*^) are shown. The statistical significance of *Q* ^*2*^ diagnostics was confirmed for all PLS models by permutation testing (*p* < 0.05; see supplementary material 9)

Unsupervised (PCA) and supervised (PLS) multivariate analyses of the whole datasets were performed, respectively, in order to have an overview of the overall variability, and to look for specific trends according to collection condition and storage duration. *Exp. 2* was analyzed first because it exhibited the highest bacterial growth (Fig. 1) and because it included the 3 collection conditions: (i) with preservative at room temperature (*Pres*), (ii) without preservative at room temperature (*RT*) and (iii) without preservative at 4 °C. Three clusters corresponding to each storage condition can be clearly observed along the second dimension of the principal component score plot modeling the positive ion mode data (Fig. 2a), despite some spread between replicates which may be due to technical variability during sample preparation and measurement. This axis seems to be negatively correlated with bacterial growth since samples kept at room temperature had the most negative values, whereas the scores of urine stored at 4 °C remained close to 0. Interestingly, samples collected with a preservative (200 mM boric acid) were grouped in a distinct cluster, which could reflect the impact of boric acid on the electrospray ionization yields in so far as no bacterial overgrowth occurred in these samples. Taken together, these results indicate that both the bacterial overgrowth and the presence of preservative impact on the metabolic composition of urine.

We further focused on the effects of the storage conditions by using partial least-square analysis (PLS) (Wold et al. 2001) (Fig. 2b). Remarkably, the time course evolution of both *RT* and *Pres* samples were correlated with the first bisector (although with a smaller variance for the latter), but not the 4 °C urines, suggesting that mechanisms other than bacterial growth (e.g. chemical degradation) impact on metabolite concentrations at room temperature. The same trends were seen with the data recorded in the negative ion mode of *Exp.* *2* (figure provided as supplementary material 7) and also with the data obtained with *Exp.* *1* (Fig. 2c, d). Only the negative mode of *Exp.* *1* did not provide significant PLS modelling, suggesting that time dependence may be masked by other sources of variability in this particular dataset. In conclusion, a discrimination between the samples stored at room temperature and those stored at 4 °C is evidenced in both experiments.

To further characterize the human urine metabolites impacted by collection conditions, we focused on the molecules that were identified by using our spectral database, on the basis of accurate mass, chromatographic retention times and MS/MS (Roux et al. 2012). Multivariate analyses on these reduced datasets showed similar patterns compared with the original data (figure provided as supplementary material 8), suggesting that restriction to identified metabolites did not introduce any major bias.

### Identification of metabolites whose concentrations are impacted by collection and storage conditions

**Fig. 3:**
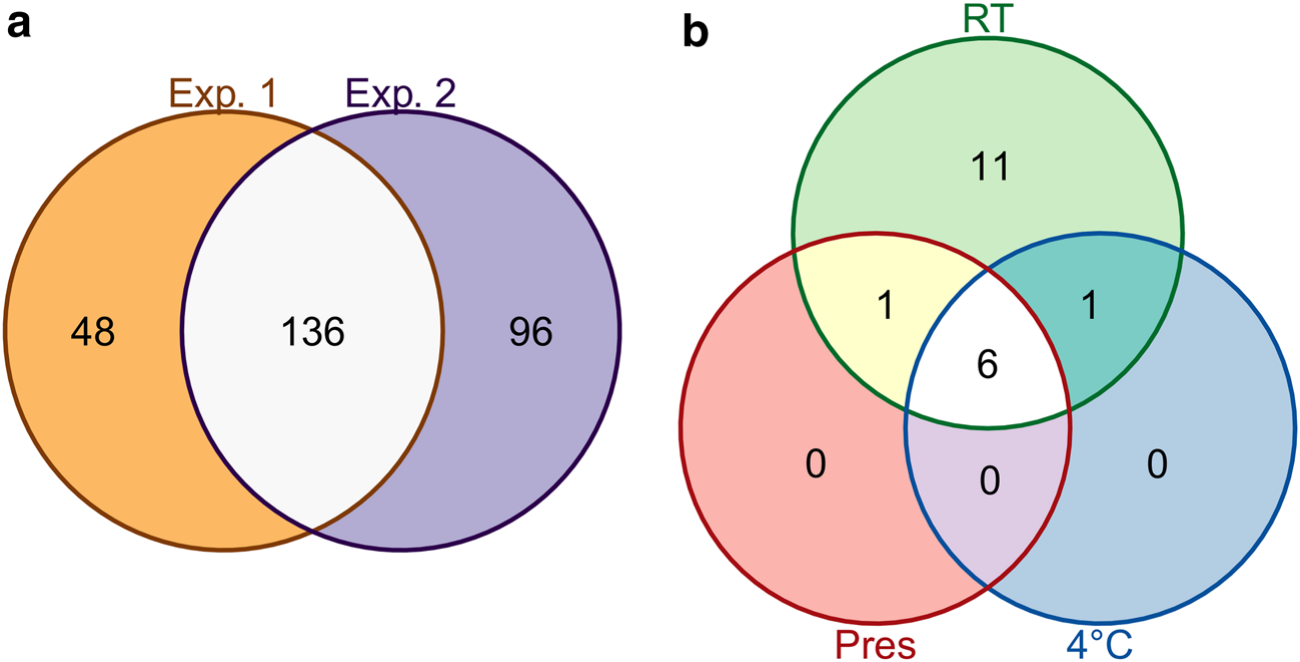
Number of metabolites impacted by storage duration. **a** Total number of identified metabolites in *Exp.* *1* and *Exp.* *2* which were used for statistical analysis. **b** Number of metabolites significantly correlated with storage duration at 4 °C or at room temperature with (*Pres*) or without (*RT*) a preservative

We identified 184 and 232 metabolites in samples from *Exp.* *1* and *Exp.* *2* respectively, including 136 that were common to both experiments (Fig. 3a). Univariate correlations between metabolite intensities and storage duration (from 0 to 72 h) were tested for each condition: *RT*, 4 °C, and *Pres* (the peak tables with chemical and statistical annotations are provided as supplementary materials 3 and 4).

We found 16 metabolites meeting the two criteria of interest: correlation with storage duration in the *RT* samples, and identical statistical significance between the two experiments (when available) in both the *RT* and 4 °C conditions (Fig. 3b). Three additional metabolites (i.e., threonolactone, and two isomers of hydroxyretinoic and ketoretinoic acid glucuronides) were also included due to their interesting concentration trends. Importantly, these univariate selection results were confirmed by multivariate PLS modeling (i.e., computation of “Variable Importance in Projection”, VIP): an average of 81 % identity between the metabolites with the lowest *p* values and the highest VIPs was obtained, and none of the few metabolites with high VIP but not significant *p* value displayed variations fully compatible with the robust selection criteria described previously. The details of the molecule names, the variation trends and the first time point with concentrations all above (or below, depending on the trend) T_0_ are reported in Table 2. For the majority of the metabolites, the impact of sample collection and storage conditions on urinary metabolite concentrations was observed by 24 h (with triplicate concentrations all higher, or lower, than at T_0_).

**Table 2:**
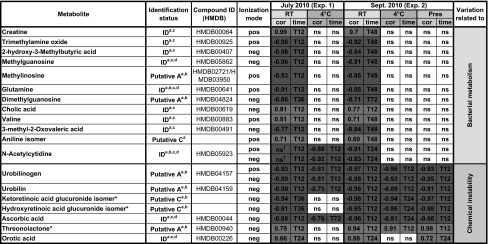
Effect of storage conditions on metabolite concentration variation over time.

Metabolites whose concentrations were impacted by either bacterial metabolism or chemical instability were sorted by increasing corrected *p*-values associated with correlation with room temperature, 4 °C and preservative storage duration. Metabolites were identified according to the metabolomics standard initiative (MSI) criteria (Sumner et al 2007)

*cor* The value of the spearman correlation coefficient when the FDR of the test is <0.01 (ns otherwise) is colored in *green*/*red* according to its sign; *time*: first time point at which triplicate concentrations are all above (in case of positive correlation, below otherwise) the T_0_ triplicate concentrations. *ID*: identified (i.e., level 1); *Putative A*: putatively annotated compound (i.e., level 2); *Putative C*: putatively characterized compound (i.e., level 3)

^a^ m/z matching structure

^b^ MS^n^ matching structure

^c^ retention times (C_18_ and PFPP chromatography columns) matching standards (Roux, 2012)

^d^ MS^n^ matching standard

* Three metabolites, although not meeting all statistical criteria at the 0.01 threshold, were included in the table because of meaningful variation trends, and are discussed in the results section

^†^The decrease of the N-acetylcytidine concentrations in *Exp 1* is so abrupt (fall to 0 as soon as T_12_; see the graphics for N-acetylcytidine in the supplementary information) that the correlation test fails to reflect this variation

By comparing results between groups, two sources of instability (i.e., bacterial overgrowth or chemical instability) were found: a significant correlation only at *RT* indicated an impact of bacterial overgrowth, whereas a significant correlation at *RT* and 4 °C, and/or with preservative indicated chemical instability (Table 2).

#### Changes in metabolite concentrations related to bacterial overgrowth

According to previously published studies, some metabolites detected by NMR have already been linked to bacterial contamination (Lauridsen et al. 2007; Maher et al. 2007; Saude and Sykes 2007), but not in the same experimental conditions as those reported in the present study. Indeed, these studies addressed metabolite stability issues related to either long-term storage for periods ranging from weeks (Saude and Sykes 2007) to months (Lauridsen et al. 2007; Maher et al. 2007), or short-term storage, but at temperatures equal to or below +4 °C (Barton et al. 2008; Maher et al. 2007). Regarding the 13 reported metabolites linked to stability issues (i.e., acetic acid, benzoic acid, creatine, creatinine, lactic acid, citric acid, hippuric acid, succinic acid, malonic acid, formic acid, trimethylamine, urea, alanine and glycine), 5 (i.e., creatine, creatinine, lactic acid, citric acid, hippuric acid, and malonic acid) were detected by our LC/MS method, and all of them except for benzoic acid were detected by NMR. The concentration variations of these 13 metabolites are provided as supplementary materials 1, 3 and 4. Of note, no other metabolite was highlighted by multivariate statistical analyses (PCA score plots of NMR results for both experiments are provided as supplementary material 2).

**Fig. 4:**
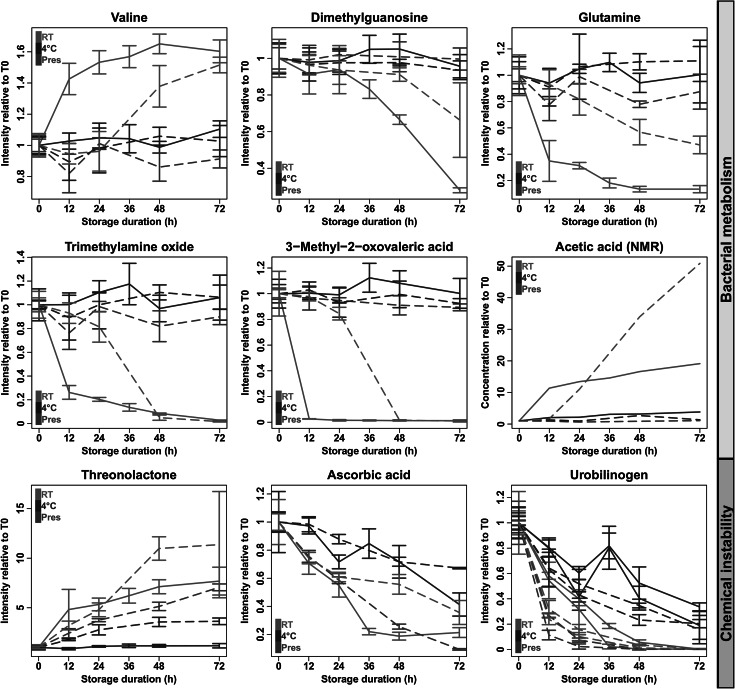
Metabolites whose concentrations are affected by bacterial overgrowth or chemical instability. Metabolite intensities were measured by LC–MS (*n* = 3) or NMR (acetic acid; *n* = 1). For each storage condition, intensities were normalized relative to the (mean of) T_0_ (triplicate) value(s). The (means of the) relative intensities are plotted as *solid lines* for *Exp.* *1* and *dashed lines* for *Exp.* *2*. In case of triplicates, standard deviations are indicated as *bars*. For urobilinogen, the *two curves* for each condition correspond to the positive and negative ionization modes

Increased concentrations of creatine observed using LC/MS and NMR were consistent with published NMR data (Saude and Sykes 2007). We did not observe any increase of creatinine or decrease of citric acid levels using either LC/MS or NMR, contrary to what has been noted in long-term stability studies using NMR experiments (Lauridsen et al. 2007; Maher et al. 2007). No clear trend was observed regarding malonic acid in our LC/MS and NMR data. The reported increase of lactic acid in long-term stability studies was only observed in *Exp.* *2* from LC/MS data, whereas concentrations of this metabolite were decreased in samples of *Exp.* *1* stored at room temperature and analyzed using both LC/MS and NMR. Finally, no significant variation was observed in our LC/MS conditions for hippuric acid, whereas concentrations increased by a factor of 1.5 were measured by NMR in those samples of *Exp.* *2* that were stored at *4* *°C*. Lastly, from our NMR data, increased concentrations of acetic acid were observed in both experiments (Fig. 4; consistent with published NMR data from long-term stability studies) whereas the levels of glycine, alanine and trimethylamine were not altered (the time courses of metabolites monitored by NMR are provided as supplementary material 1).

Furthermore, 11 metabolites whose variations related to bacterial contamination had not been described previously were observed in our LC/MS data sets: trimethylamine oxide, 2-hydroxy-3-methylbutyric acid, methylguanosine, methylinosine, glutamine, dimethylguanosine, cholic acid, valine, 3-methyl-2-oxovaleric acid, an isomer of aniline, and N-acetylcytidine (Table 2; Figure 4, and graphs provided as supplementary material 6). Some of these variations may be easily explained by bacterial fermentation, as is, for example, the case with sugar derivatives, amino acids and nucleoside derivatives (Janssens et al. 2014). In addition, decreased concentrations of trimethylamine oxide (TMAO; Fig. 4), which were also observed in NMR measurements, may be linked to bacterial overgrowth since this compound is involved in the metabolism of microorganisms.

Interestingly, concentration variations of most of these metabolites were detected from 12 h in *Exp. 1*, and rather at 48 h in *Exp. 2*. This can be explained by the differences of bacterial overgrowth kinetics observed between the two experiments: bacterial overgrowth can be observed from 12 h in *Exp. 1* whereas it is delayed in *Exp. 2* (Fig. 1).

#### Variations related to chemical stability

Seven metabolites exhibited changes in concentrations both at room temperature (without preservative) and with preservative or at 4 °C: urobilinogen, urobilin, ascorbic acid, orotic acid, in addition to isomers of ketoretinoic acid and hydroxyretinoic acid glucuronides, and threonolactone (Table 2; Figure 4, and graphs provided as supplementary material 6). The three latter metabolites were included into the list because, although the correlation significances at 4 °C differed between *Exp.* *1* and *Exp.* *2* at the 0.01 FDR threshold, the trends were similar. As no bacterial contamination was observed with preservative or at 4 °C, these variations cannot be explained by bacterial overgrowth, but rather by chemical instability in solution. Concentration variations were more extreme in *RT* conditions than at 4 °C, as observed for the decrease of ascorbic acid levels (Fig. 4). The latter was confirmed by increases in concentration of threonolactone, the autoxidation product of ascorbic acid, in both *RT* and *Pres* samples (Fig. 4).

## Concluding remarks

We highlighted here the metabolic consequences of bacterial contamination of urine samples, and also the chemical instability of some metabolites. Among the 280 detected metabolites, 19 (i.e. 7 %) showed robust significant changes in concentration according to storage conditions, whether due to bacterial contamination (12/19) or chemical instability in solution (7/19). In addition to metabolites already known to be affected by bacterial overgrowth, such as creatine and acetic acid, other metabolites were shown to be impacted by bacterial metabolism, such as those related to the metabolism of nucleosides, amino acids, or choline. Chemical instability in solution was observed to a lesser extent, and was also highly correlated with the storage temperature.

Sampling conditions therefore have an impact on the chemical composition of urine. Most changes occur at room temperature from 12 h onwards and are depending on kinetics of bacterial overgrowth. Preservatives are effective in preventing bacterial contamination, but not in avoiding chemical instability, contrary to collection on ice which both prevents bacterial growth and limits metabolite degradation. Our results obtained on human urine are consistent with GC/MS data on rodent urine (Bando et al. 2010) showing that collection in metabolism cages at 4 °C limits changes in metabolite content of urine samples and demonstrate that collection on ice is the most effective sampling condition.

## Electronic supplementary material

Below is the link to the electronic supplementary material.

Supplementary material 1 (XLSX 14 kb)

Supplementary material 2 (PPTX 90 kb)

Supplementary material 3 (XLSX 159 kb)

Supplementary material 4 (XLSX 235 kb)

Supplementary material 5 (PDF 88 kb)

Supplementary material 6 (ZIP 97 kb)

Supplementary material 7 (TIFF 8613 kb)

Supplementary material 8 (TIFF 17227 kb)

Supplementary material 9 (TIFF 13535 kb)
