# Supplementary material for: Impact of collection conditions on the metabolite content of human urine samples as analyzed by liquid chromatography coupled to mass spectrometry and nuclear magnetic resonance spectroscopy
Source: Metabolomics. 2014 Dec 23;11(5):1095–105. doi: 10.1007/s11306-014-0764-5 (PMC4559108; doi:10.1007/s11306-014-0764-5)
Supplement: Supplementary file 2 — Supplementary material 2 (PPTX 90 kb) [file 11306_2014_764_MOESM2_ESM.pptx]

## Slide 1
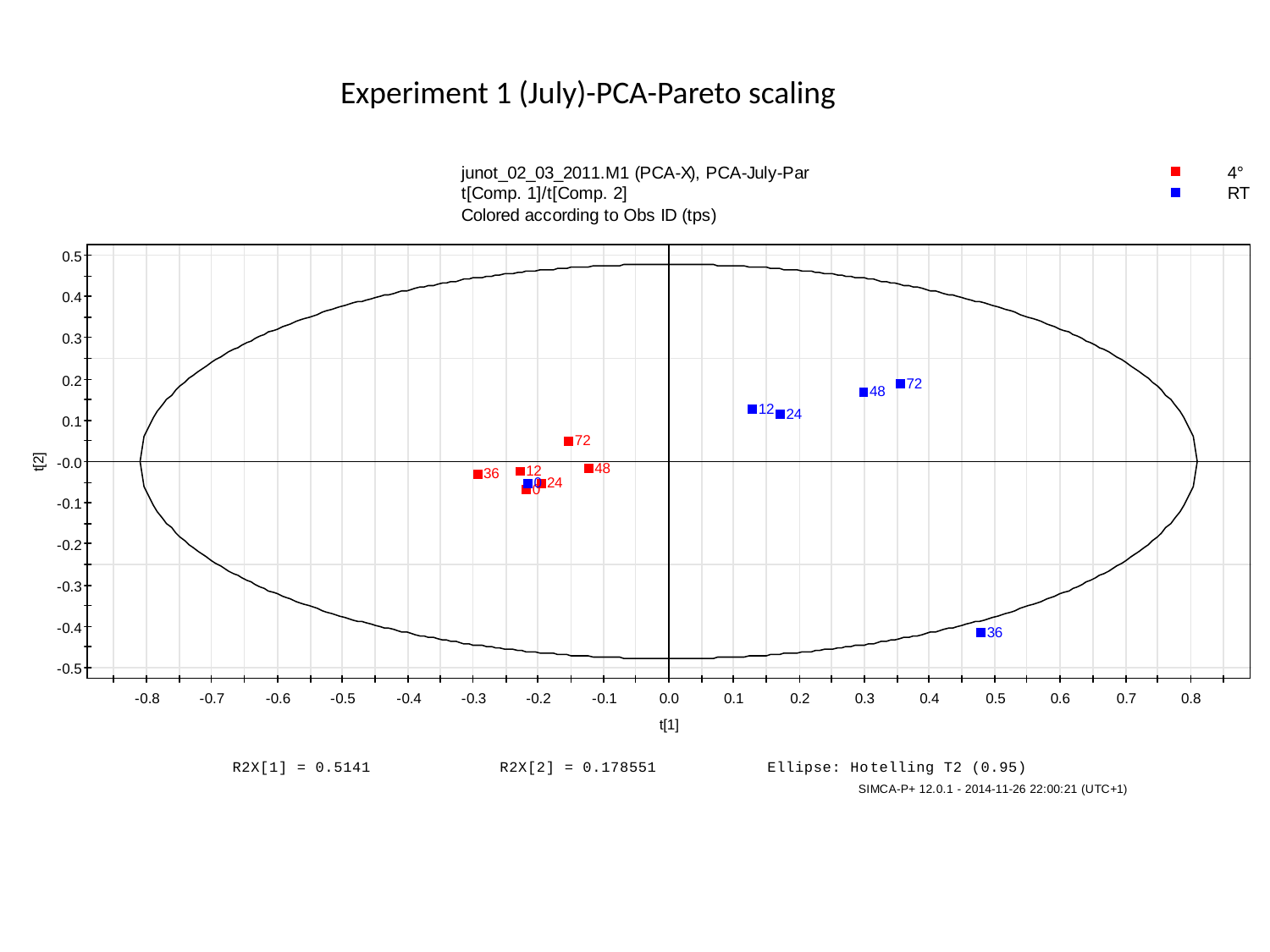

Experiment 1 (July)-PCA-Pareto scaling

## Slide 2
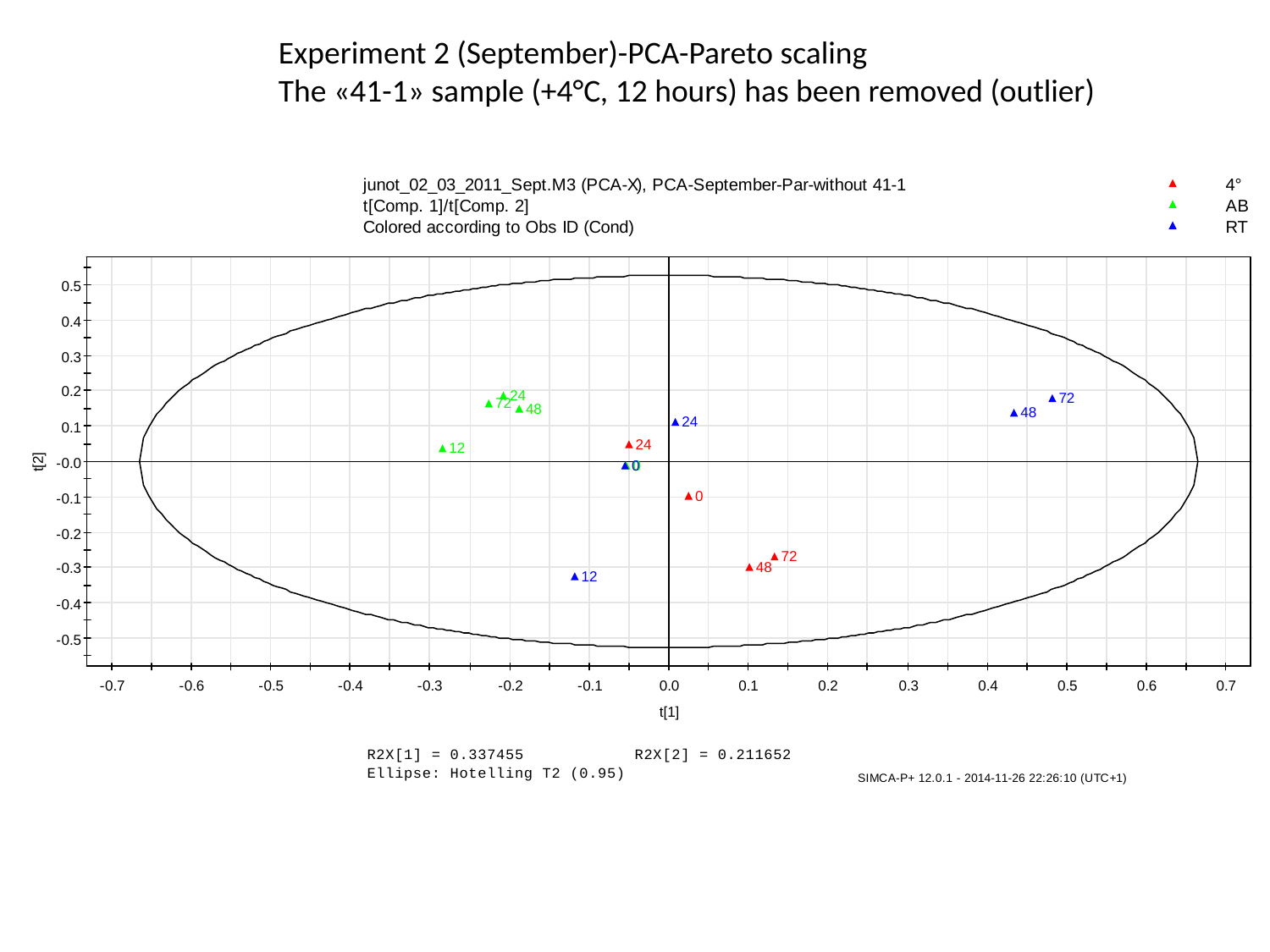

Experiment 2 (September)-PCA-Pareto scaling
The «41-1» sample (+4°C, 12 hours) has been removed (outlier)
