# Supplementary material for: Impact of collection conditions on the metabolite content of human urine samples as analyzed by liquid chromatography coupled to mass spectrometry and nuclear magnetic resonance spectroscopy
Source: Metabolomics. 2014 Dec 23;11(5):1095–105. doi: 10.1007/s11306-014-0764-5 (PMC4559108; doi:10.1007/s11306-014-0764-5)
Supplement: Supplementary file 5 — Supplementary material 5 (PDF 88 kb) [file 11306_2014_764_MOESM5_ESM.pdf]

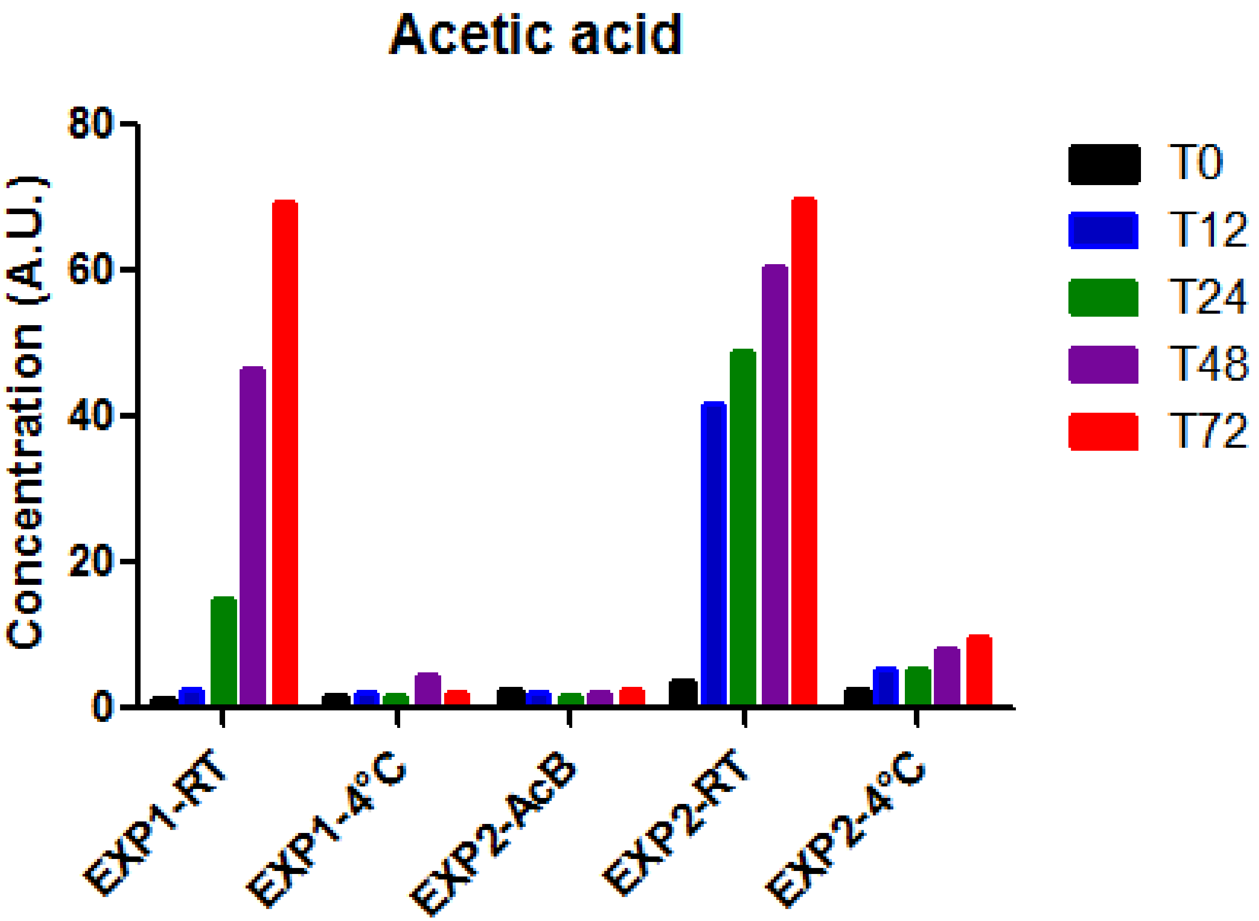

Acetic acid
Concentration (A.U.)
0
20
40
60
80
EXP1-RT
EXP1-4°C
EXP2-AcB
EXP2-RT
EXP2-4°C
T0
T12
T24
T48
T72

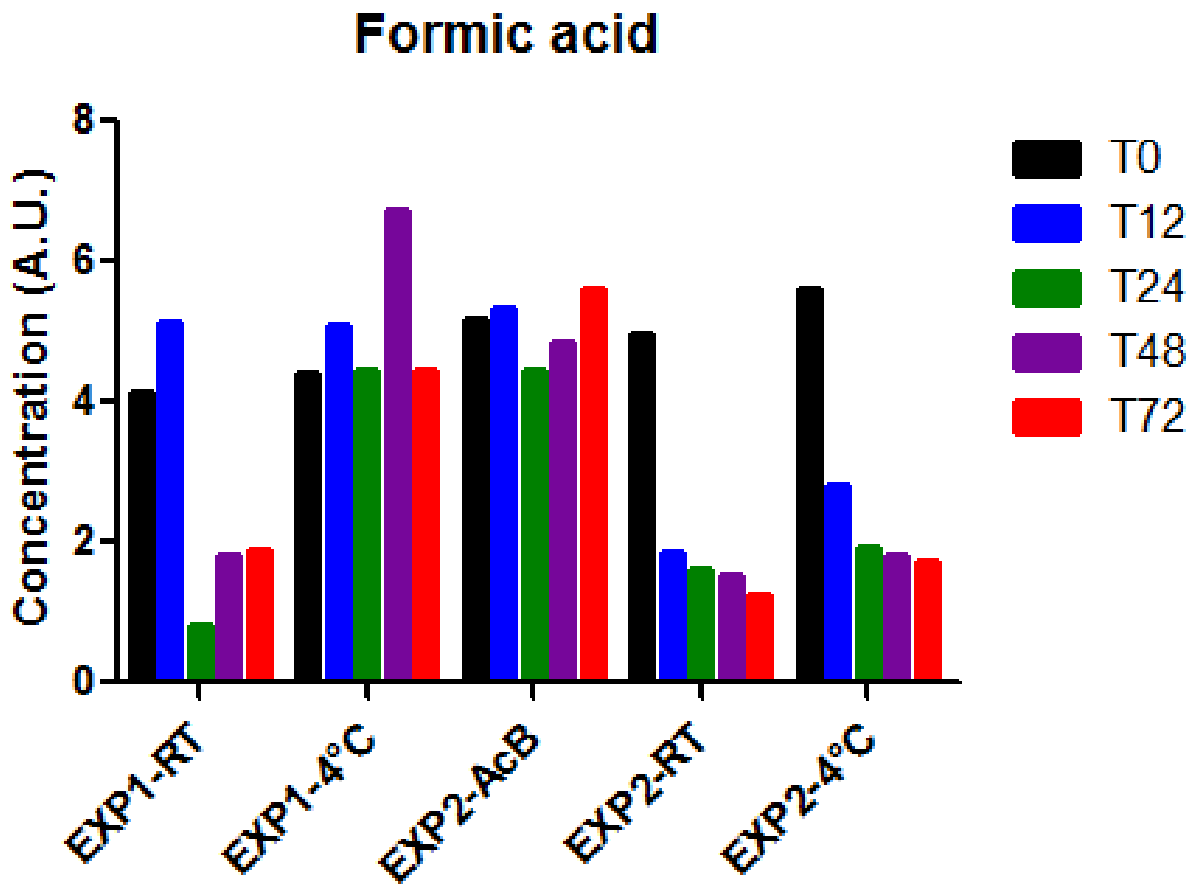
Formic acid
Concentration (A.U.)
8
6
4
2
0
EXP1-RT
EXP1-4°C
EXP2-AcB
EXP2-RT
EXP2-4°C
T0
T12
T24
T48
T72

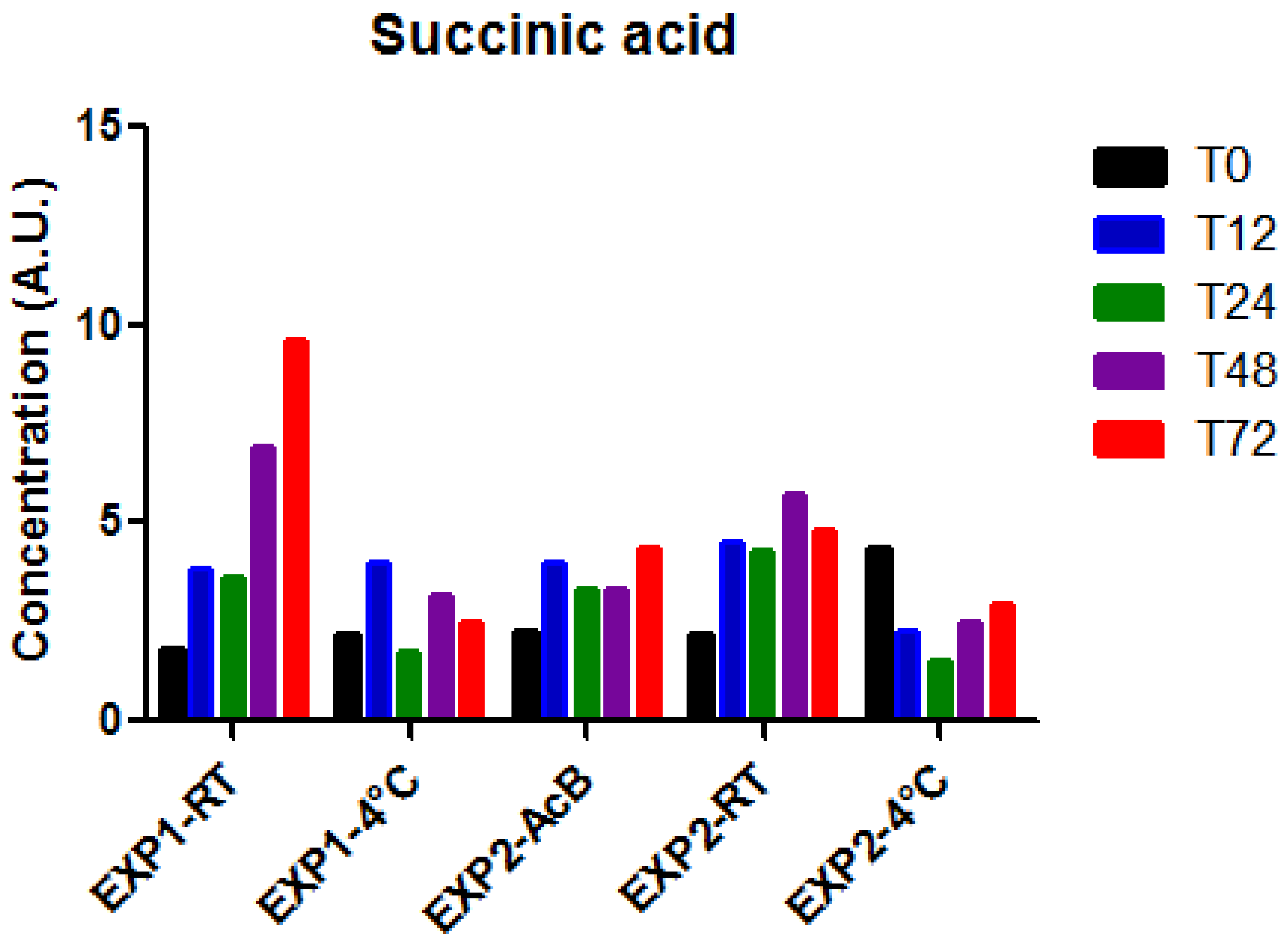

Succinic acid
Concentration (A.U.)
15
10
5
0
EXP1-RT
EXP1-4°C
EXP2-AcB
EXP2-RT
EXP2-4°C
T0
T12
T24
T48
T72

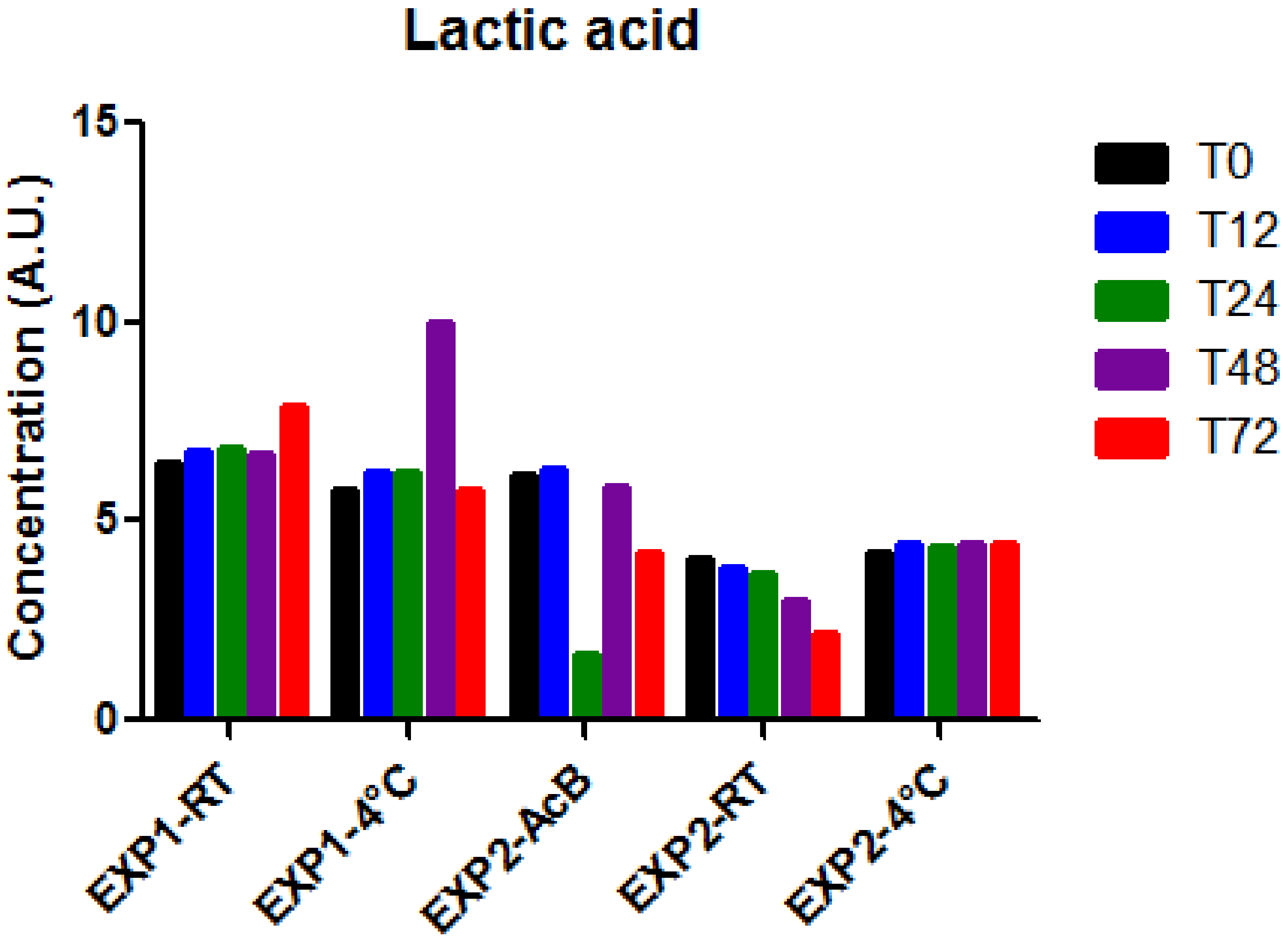

Lactic acid
Concentration (A.U.)
15
10
5
0
EXP1-RT
EXP1-4°C
EXP2-AcB
EXP2-RT
EXP2-4°C
T0
T12
T24
T48
T72

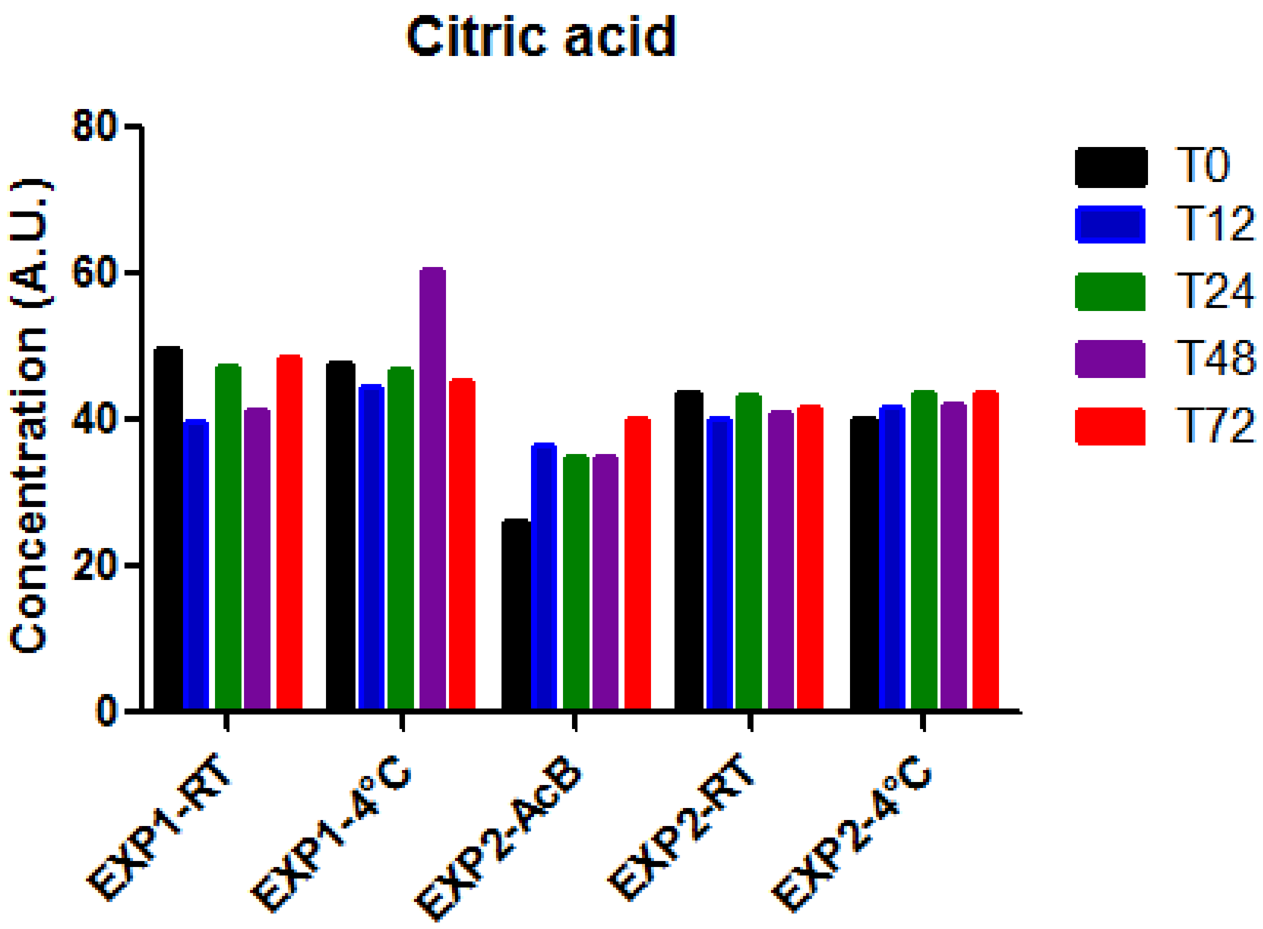

Citric acid
Concentration (A.U.)
0
20
40
60
80
EXP1-RT
EXP1-4°C
EXP2-AcB
EXP2-RT
EXP2-4°C
T0
T12
T24
T48
T72

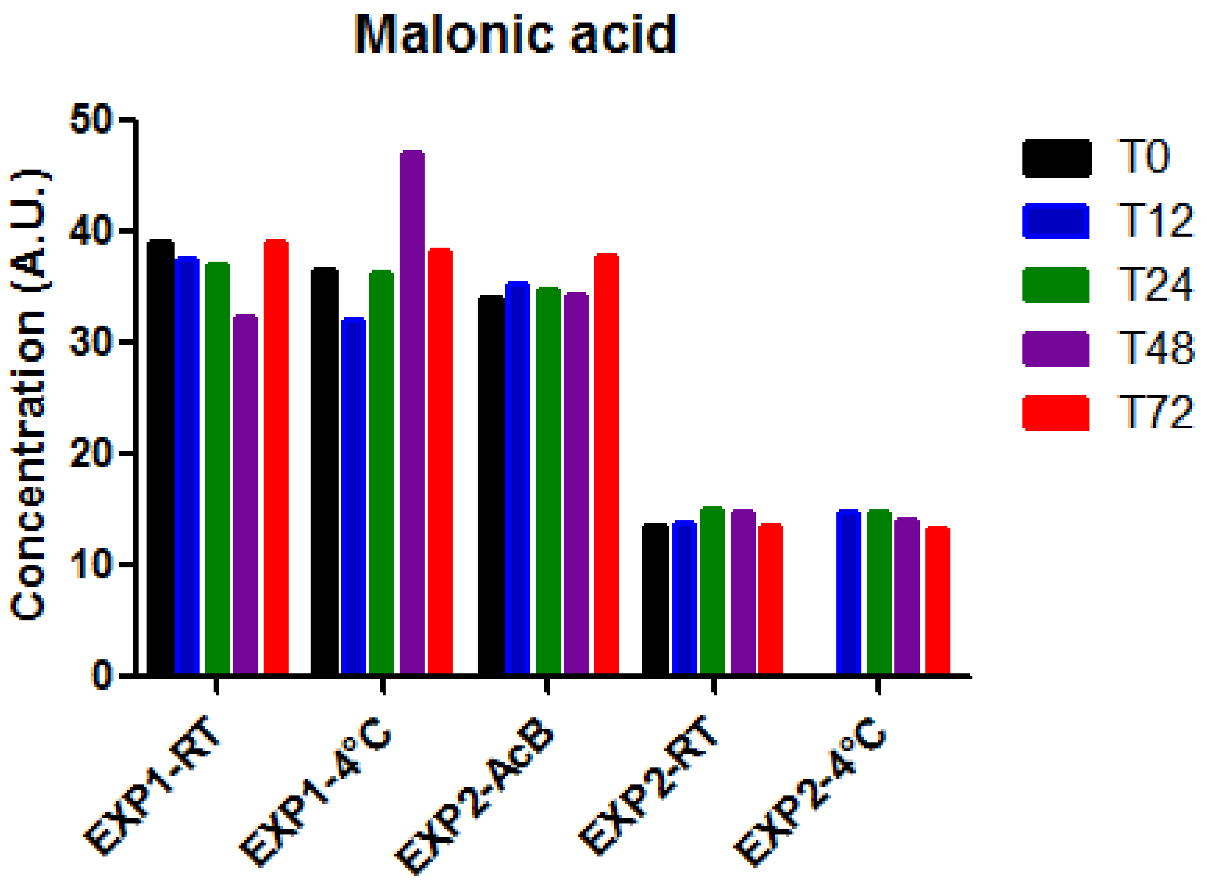

Malonic acid
Concentration (A.U.)
0
10
20
30
40
50
EXP1-RT
EXP1-4°C
EXP2-AcB
EXP2-RT
EXP2-4°C
T0
T12
T24
T48
T72

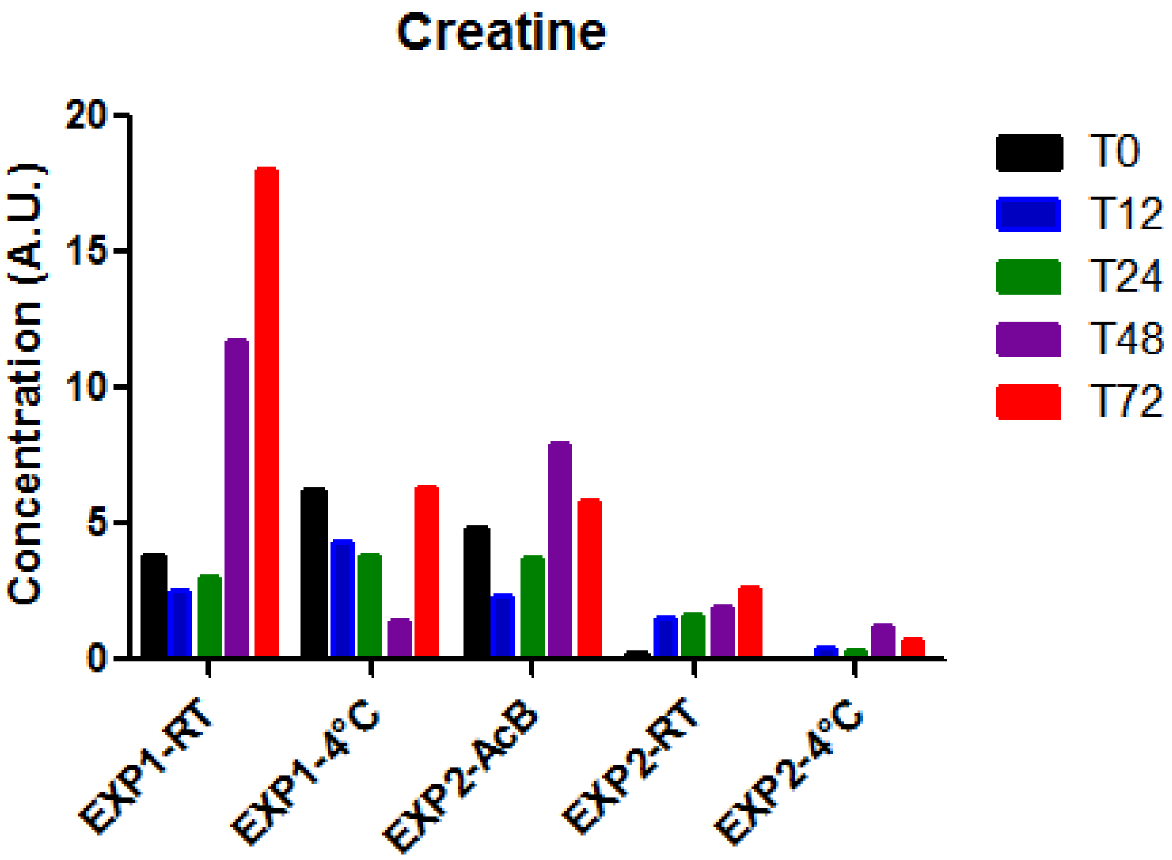
Creatine
Concentration (A.U.)
0
5
10
15
20
EXP1-RT
EXP1-4°C
EXP2-AcB
EXP2-RT
EXP2-4°C
T0
T12
T24
T48
T72

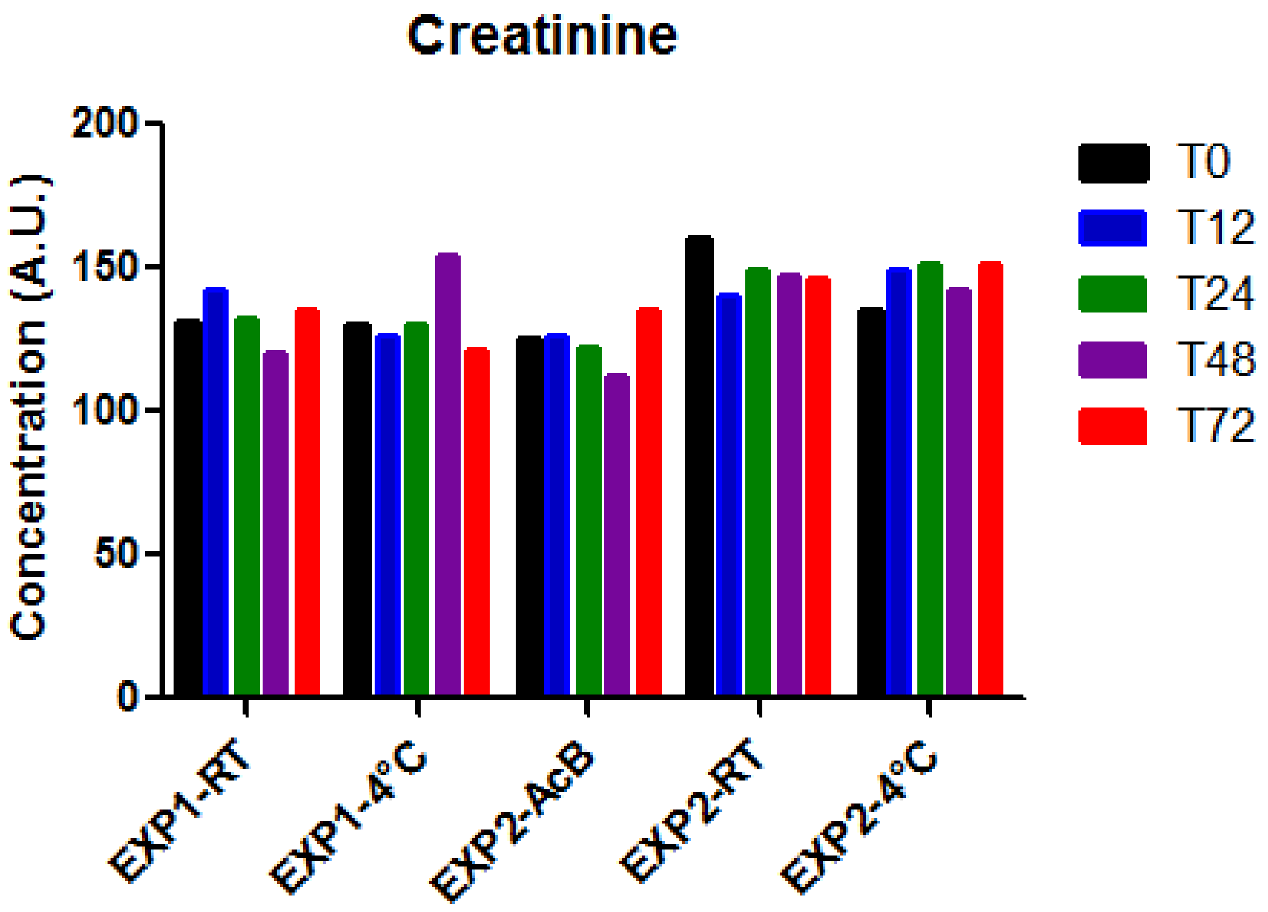
Creatinine
Concentration (A.U.)
200
150
100
50
0
EXP1-RT
EXP1-4°C
EXP2-AcB
EXP2-RT
EXP2-4°C
T0
T12
T24
T48
T72

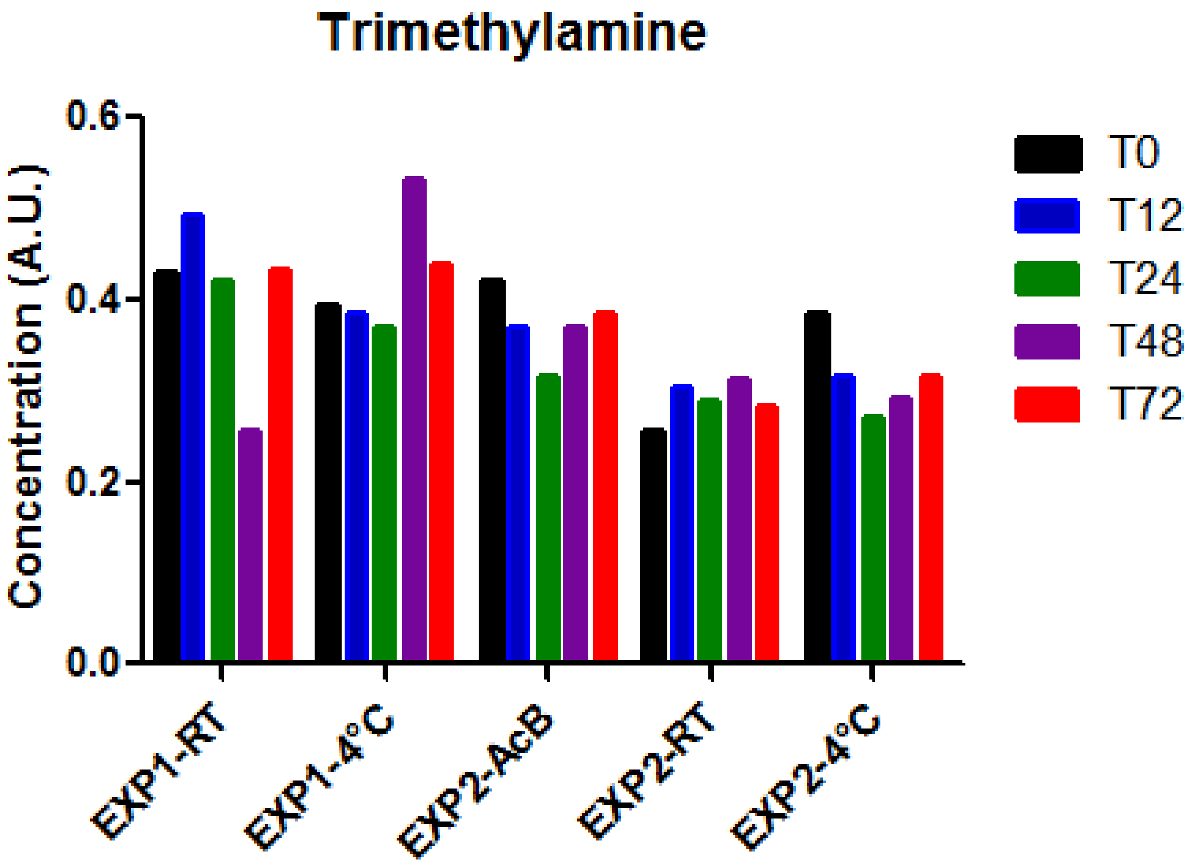
Trimethylamine
Concentration (A.U.)
0.6
0.4
0.2
0.0
EXP1-RT
EXP1-4°C
EXP2-AcB
EXP2-RT
EXP2-4°C
T0
T12
T24
T48
T72

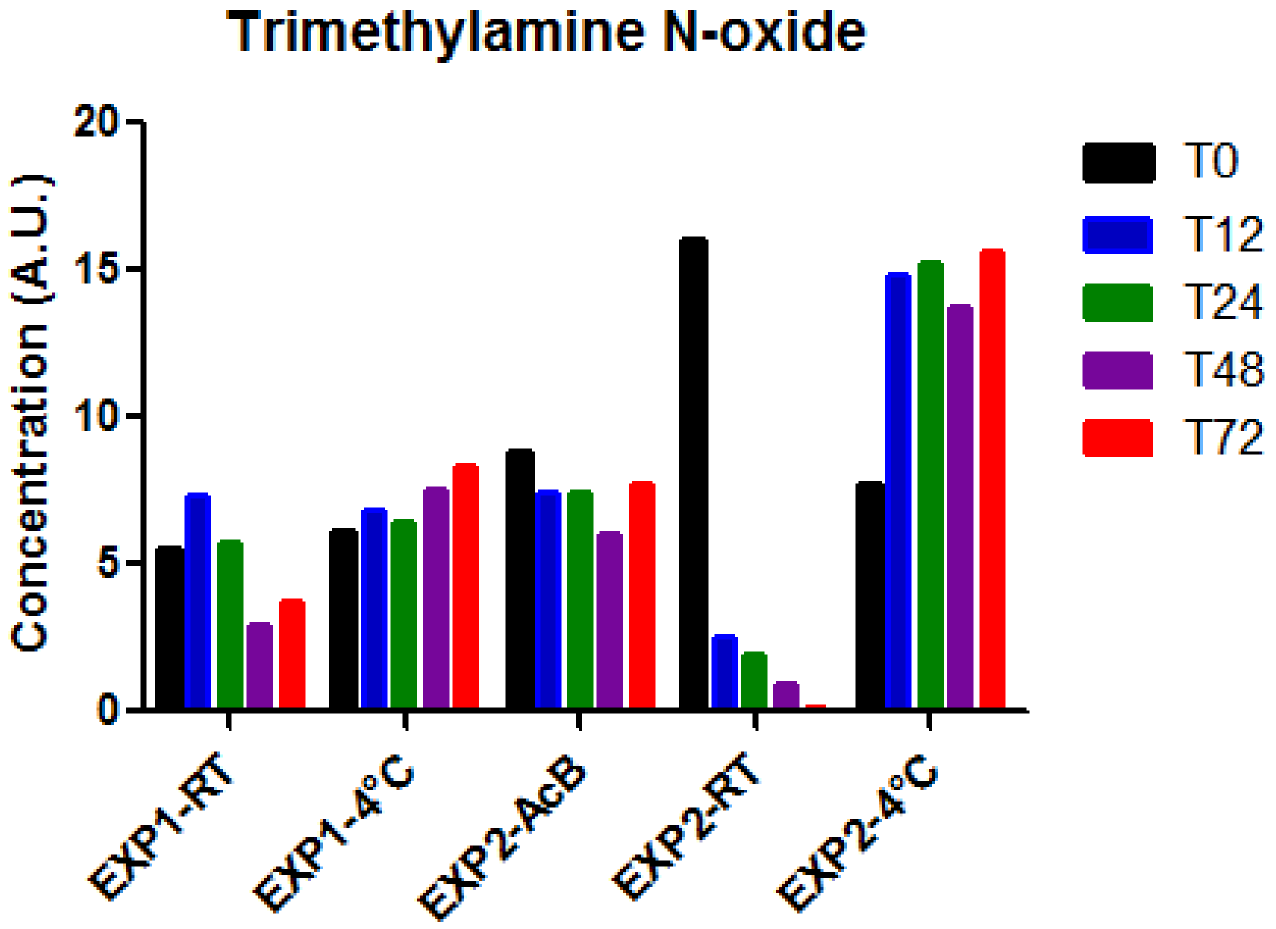
Trimethylamine N-oxide
Concentration (A.U.)
0
5
10
15
20
EXP1-RT
EXP1-4°C
EXP2-AcB
EXP2-RT
EXP2-4°C
T0
T12
T24
T48
T72

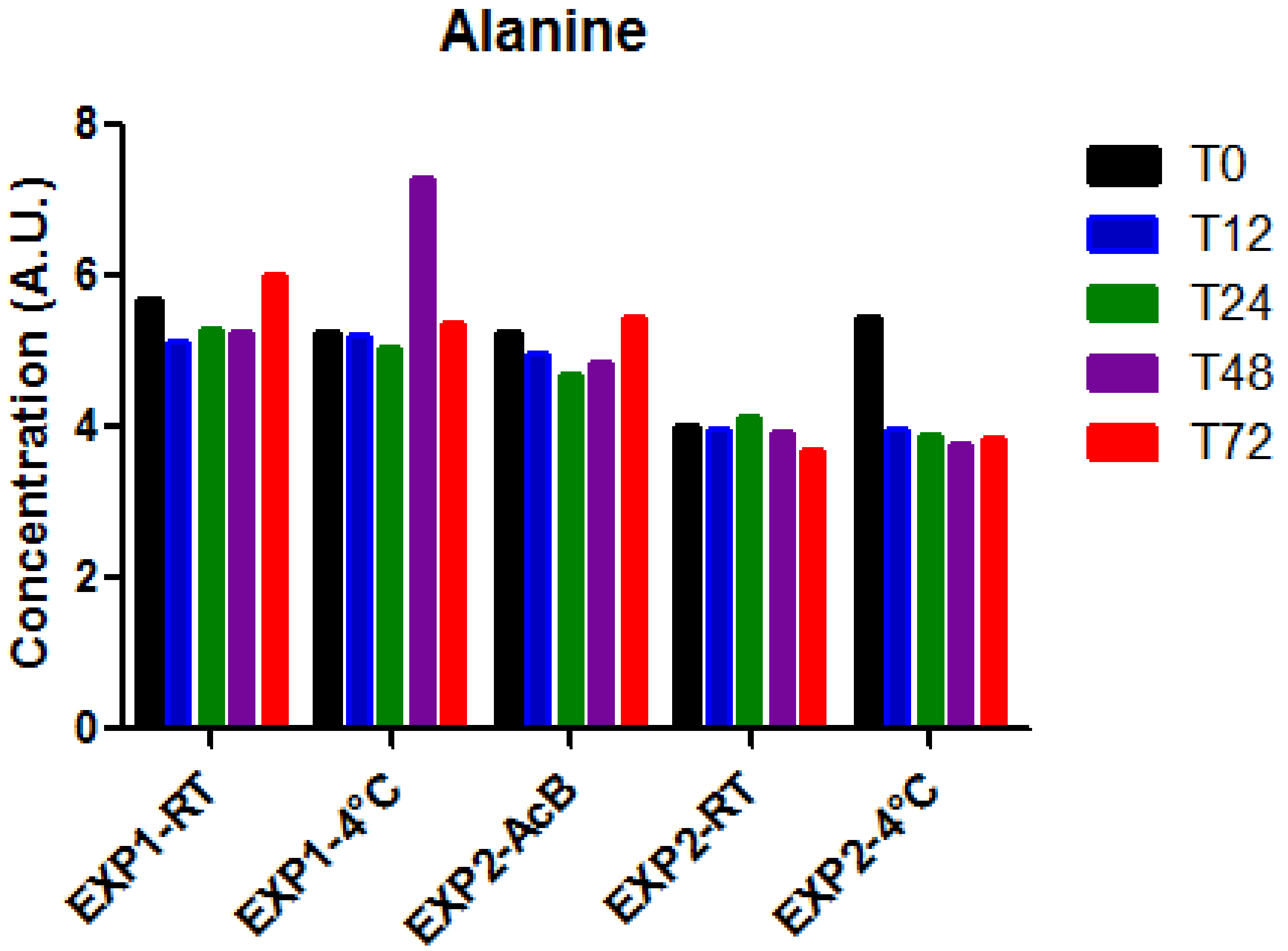
Alanine
Concentration (A.U.)
0
2
4
6
8
EXP1-RT
EXP1-4°C
EXP2-AcB
EXP2-RT
EXP2-4°C
T0
T12
T24
T48
T72

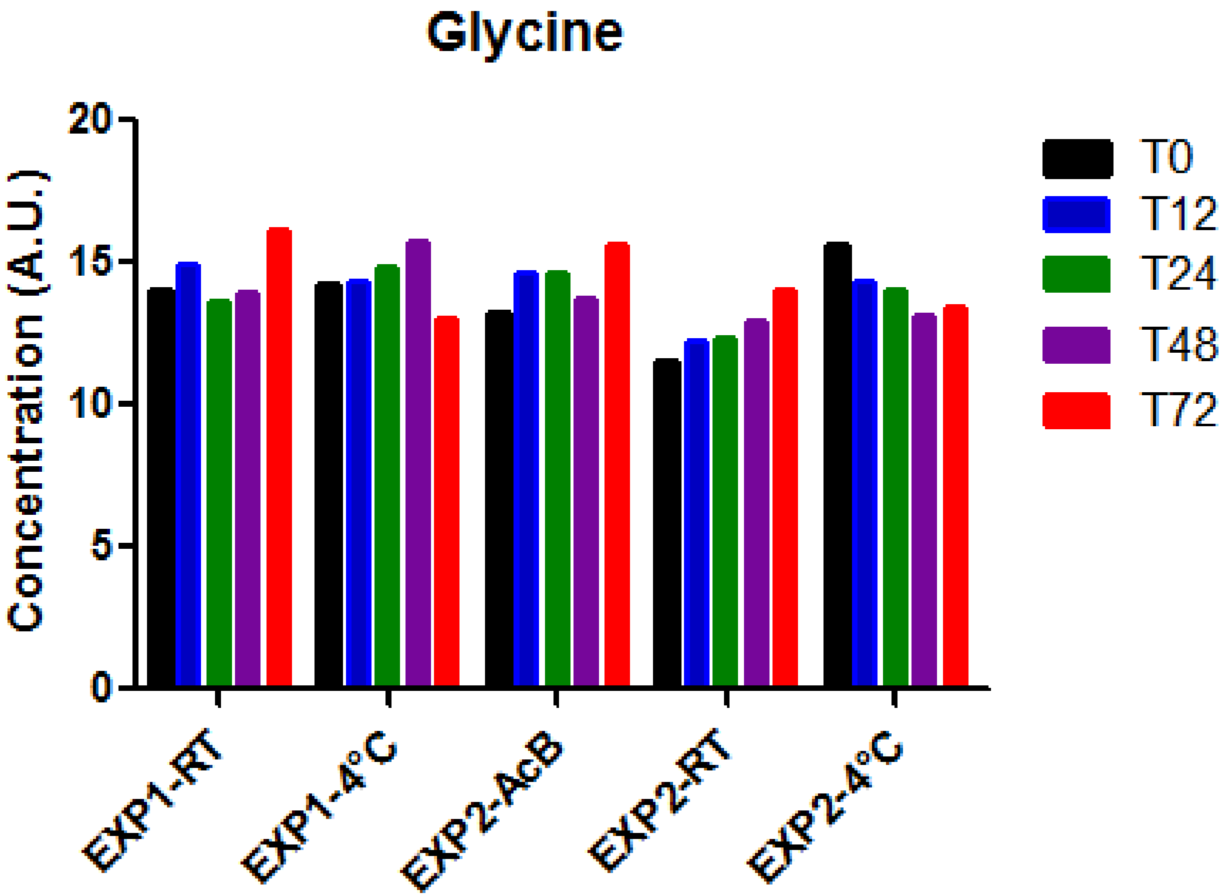
Glycine
Concentration (A.U.)
20
15
10
5
0
EXP1-RT
EXP1-4°C
EXP2-AcB
EXP2-RT
EXP2-4°C
T0
T12
T24
T48
T72

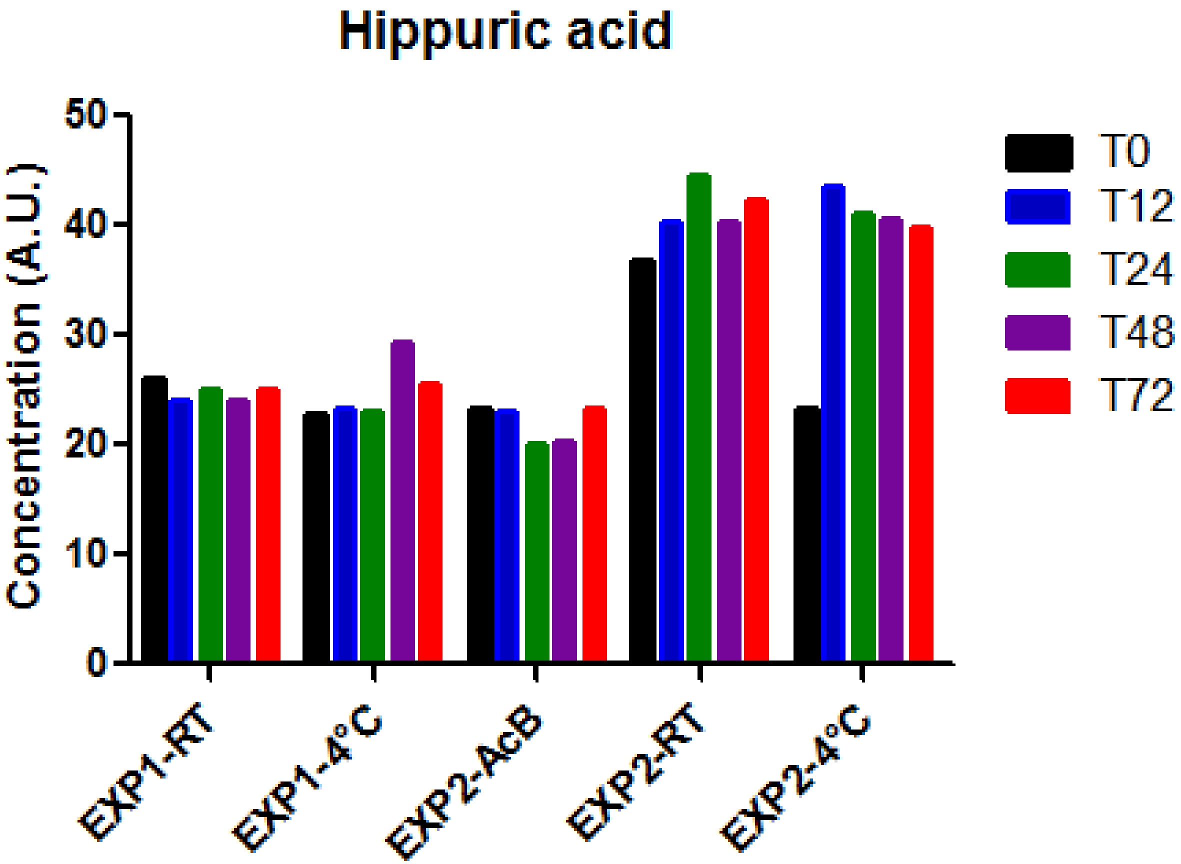

Hippuric acid
Concentration (A.U.)
0
10
20
30
40
50
EXP1-RT
EXP1-4°C
EXP2-AcB
EXP2-RT
EXP2-4°C
T0
T12
T24
T48
T72
