# Supplementary figures and images for: Impact of collection conditions on the metabolite content of human urine samples as analyzed by liquid chromatography coupled to mass spectrometry and nuclear magnetic resonance spectroscopy
Source: Metabolomics. 2014 Dec 23;11(5):1095–105. doi: 10.1007/s11306-014-0764-5 (PMC4559108; doi:10.1007/s11306-014-0764-5)

## 2-Hydroxy-3-methylbutyric acid

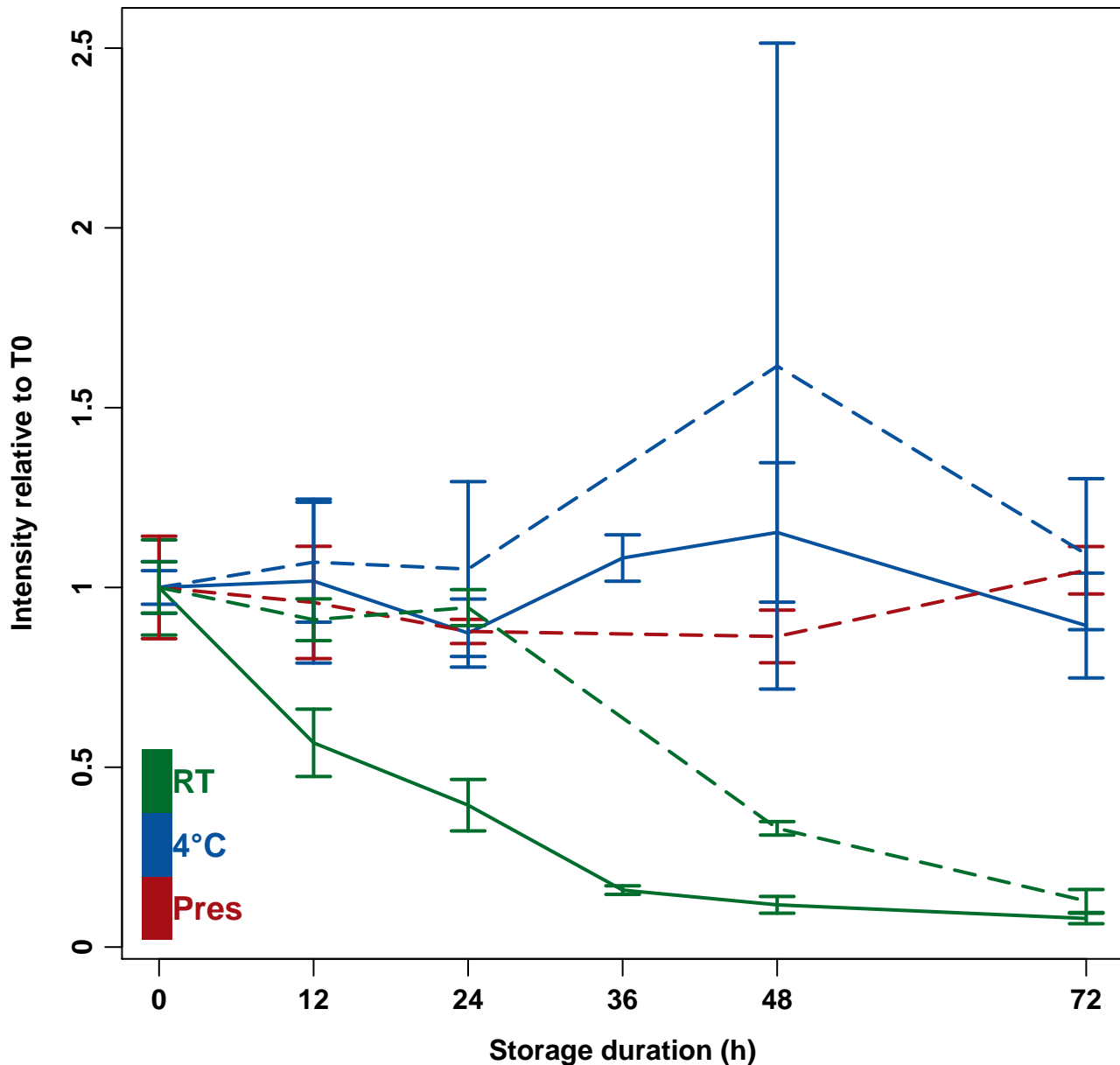

Supplement: Supplementary file 6 — Supplementary material 6 (ZIP 97 kb) [file 11306_2014_764_MOESM6_ESM.zip › 2-hydroxy-3-Methylbutyric_acid.pdf]

### 3-Methyl-2-oxovaleric acid

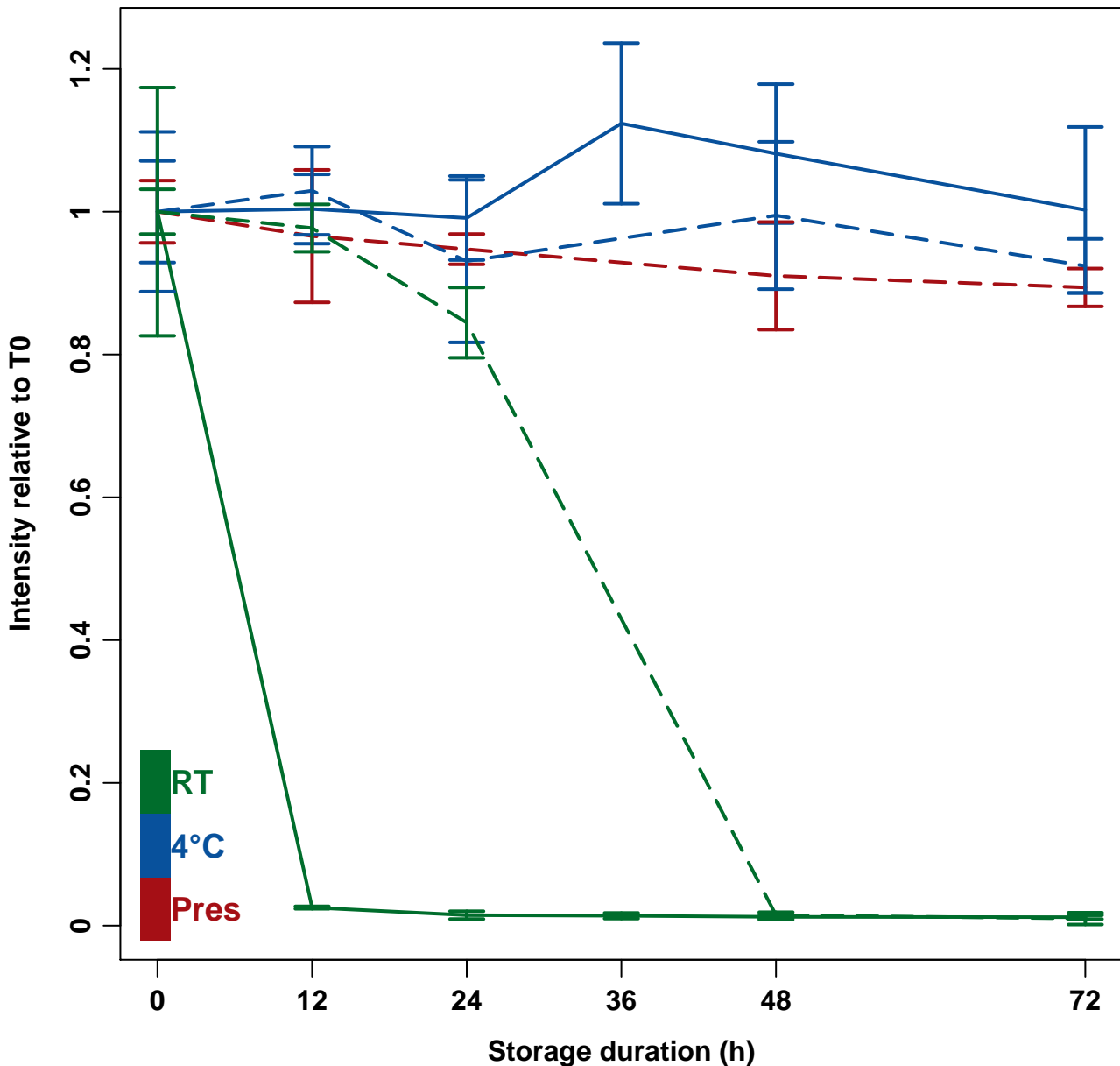

Supplement: Supplementary file 6 — Supplementary material 6 (ZIP 97 kb) [file 11306_2014_764_MOESM6_ESM.zip › 3-methyl-2-Oxovaleric_acid.pdf]

# Aniline isomer

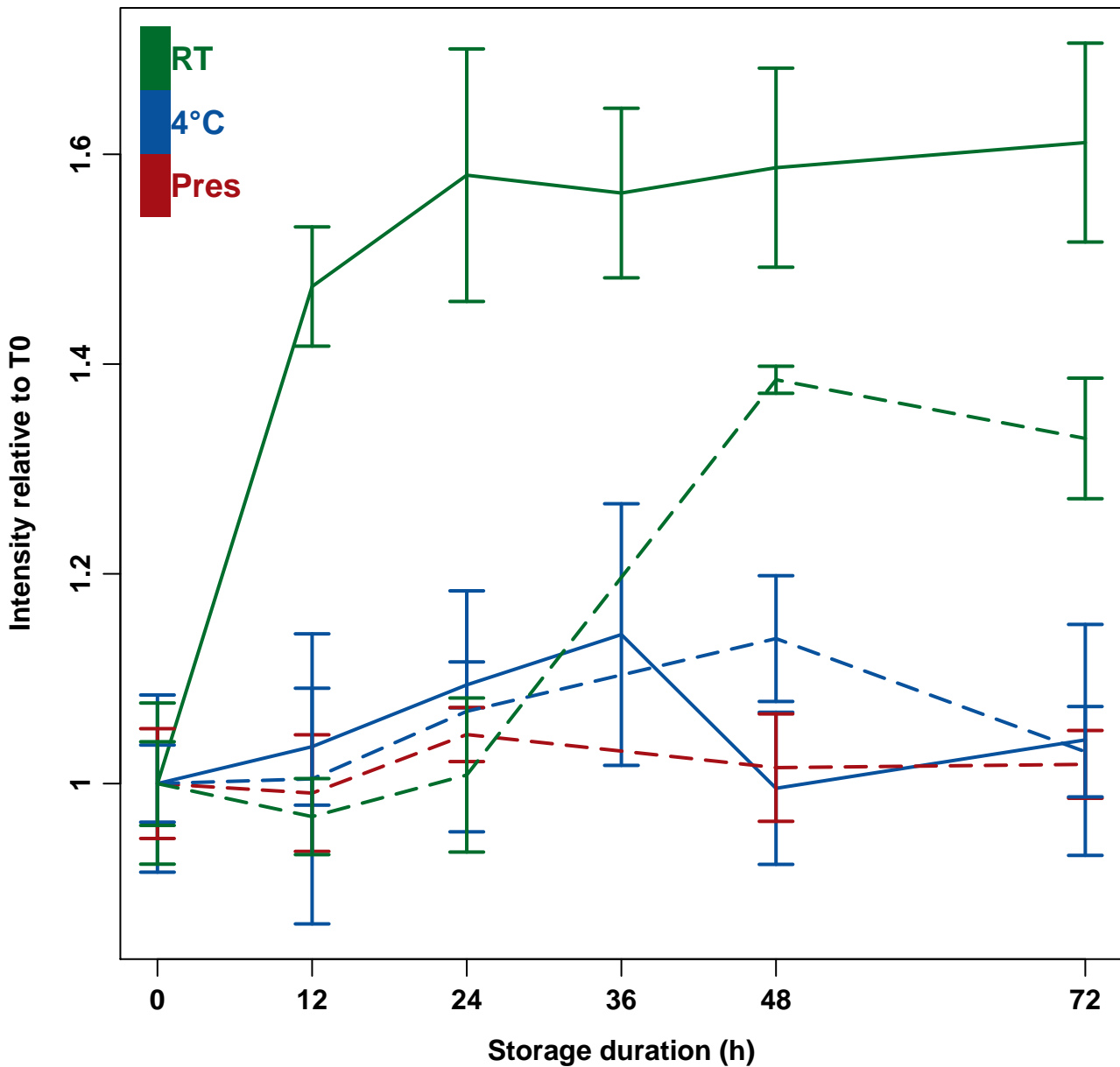

Supplement: Supplementary file 6 — Supplementary material 6 (ZIP 97 kb) [file 11306_2014_764_MOESM6_ESM.zip › Aniline_isomer.pdf]

## Ascorbic acid

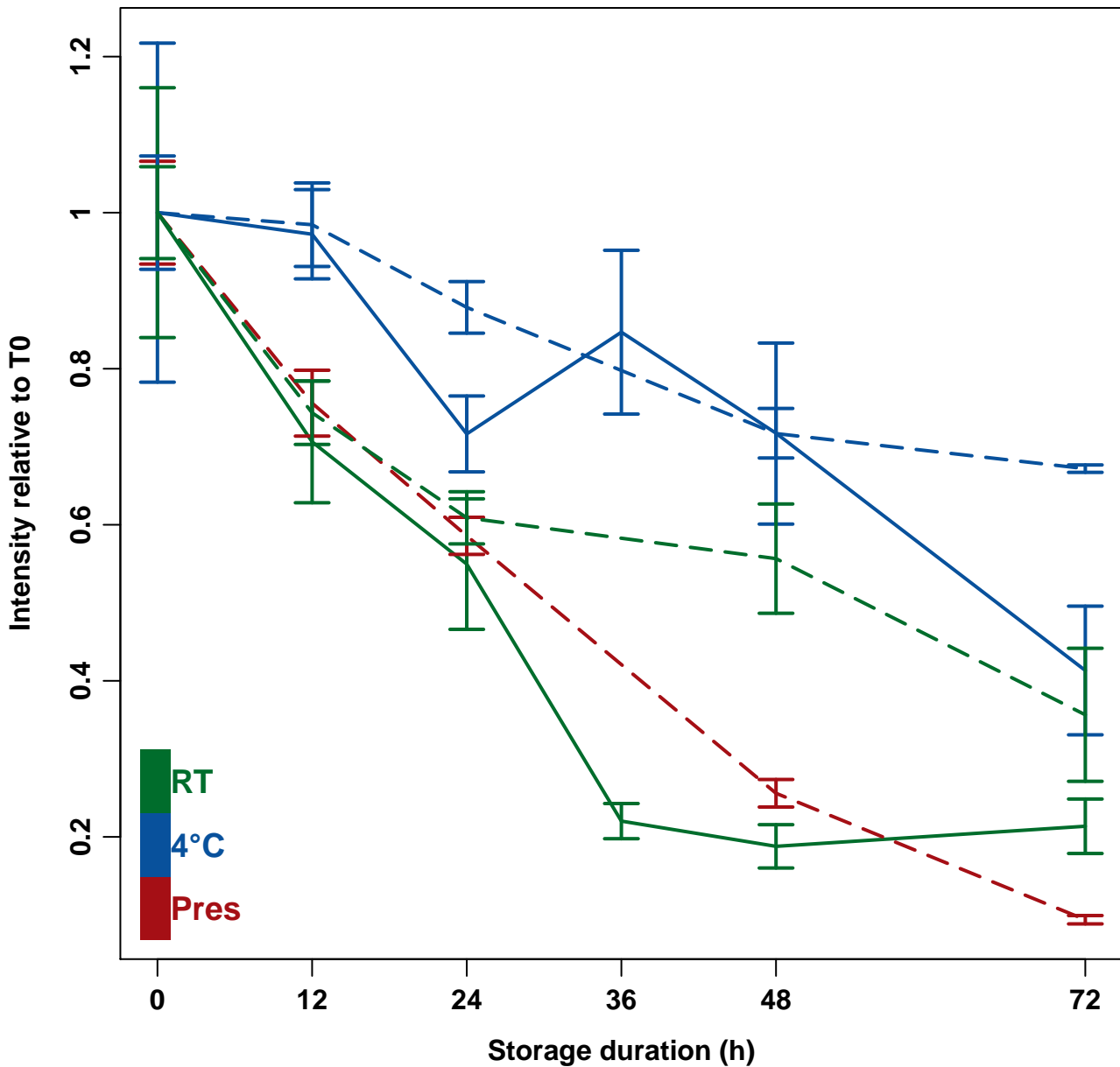

Supplement: Supplementary file 6 — Supplementary material 6 (ZIP 97 kb) [file 11306_2014_764_MOESM6_ESM.zip › Ascorbic_acid.pdf]

# Cholic acid

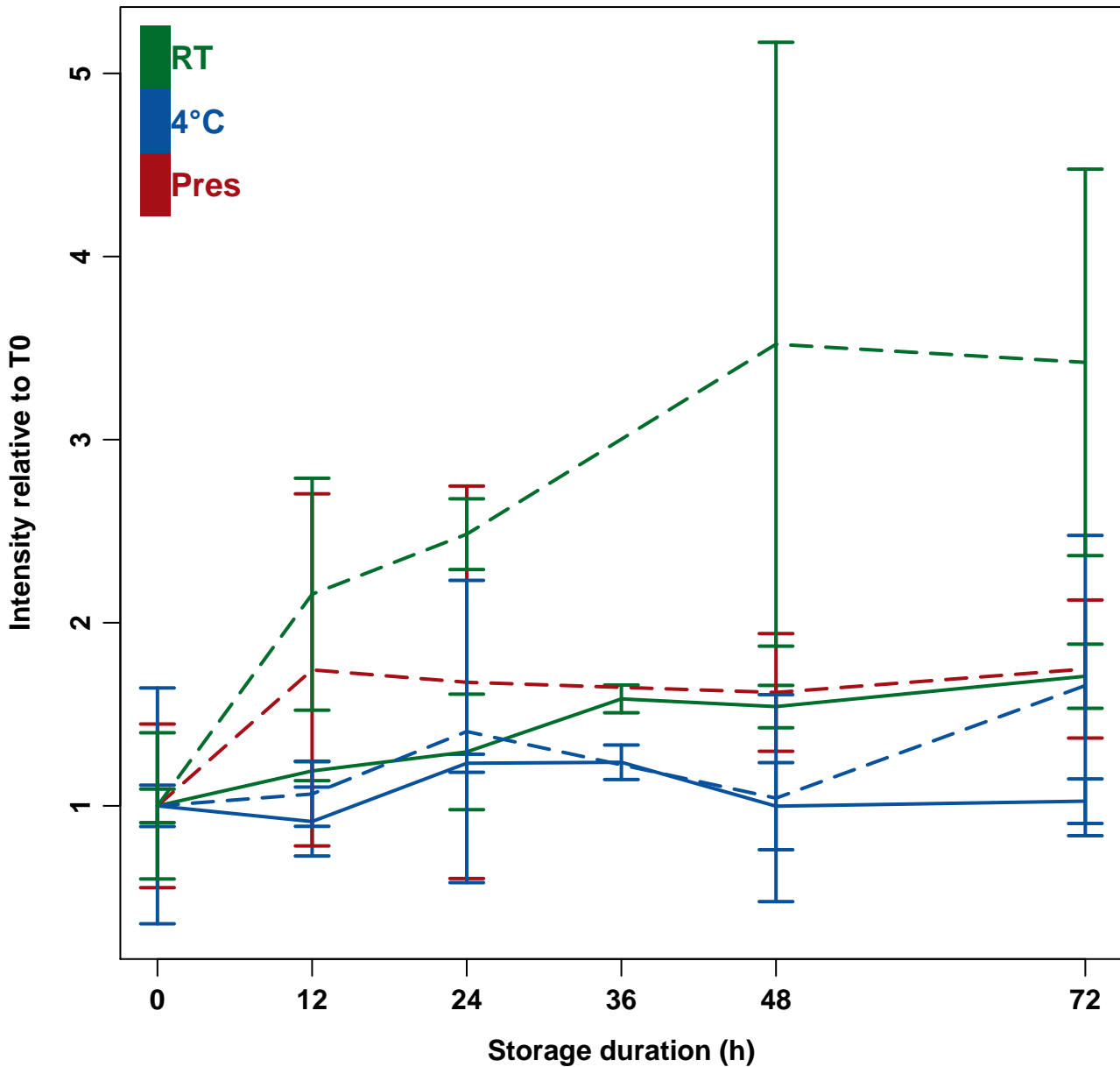

Supplement: Supplementary file 6 — Supplementary material 6 (ZIP 97 kb) [file 11306_2014_764_MOESM6_ESM.zip › Cholic_acid.pdf]

# Creatine

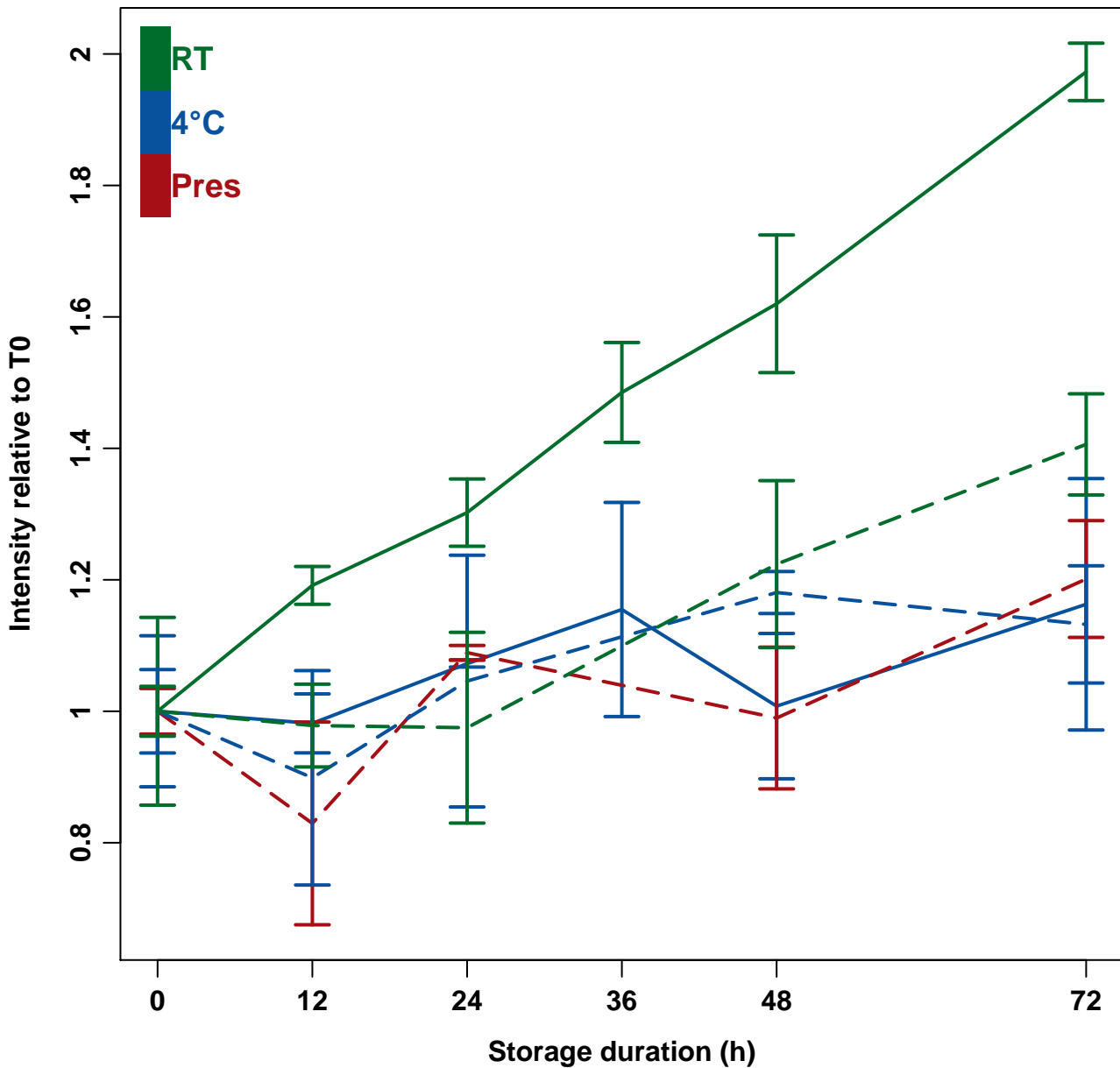

Supplement: Supplementary file 6 — Supplementary material 6 (ZIP 97 kb) [file 11306_2014_764_MOESM6_ESM.zip › Creatine.pdf]

# Dimethylguanosine

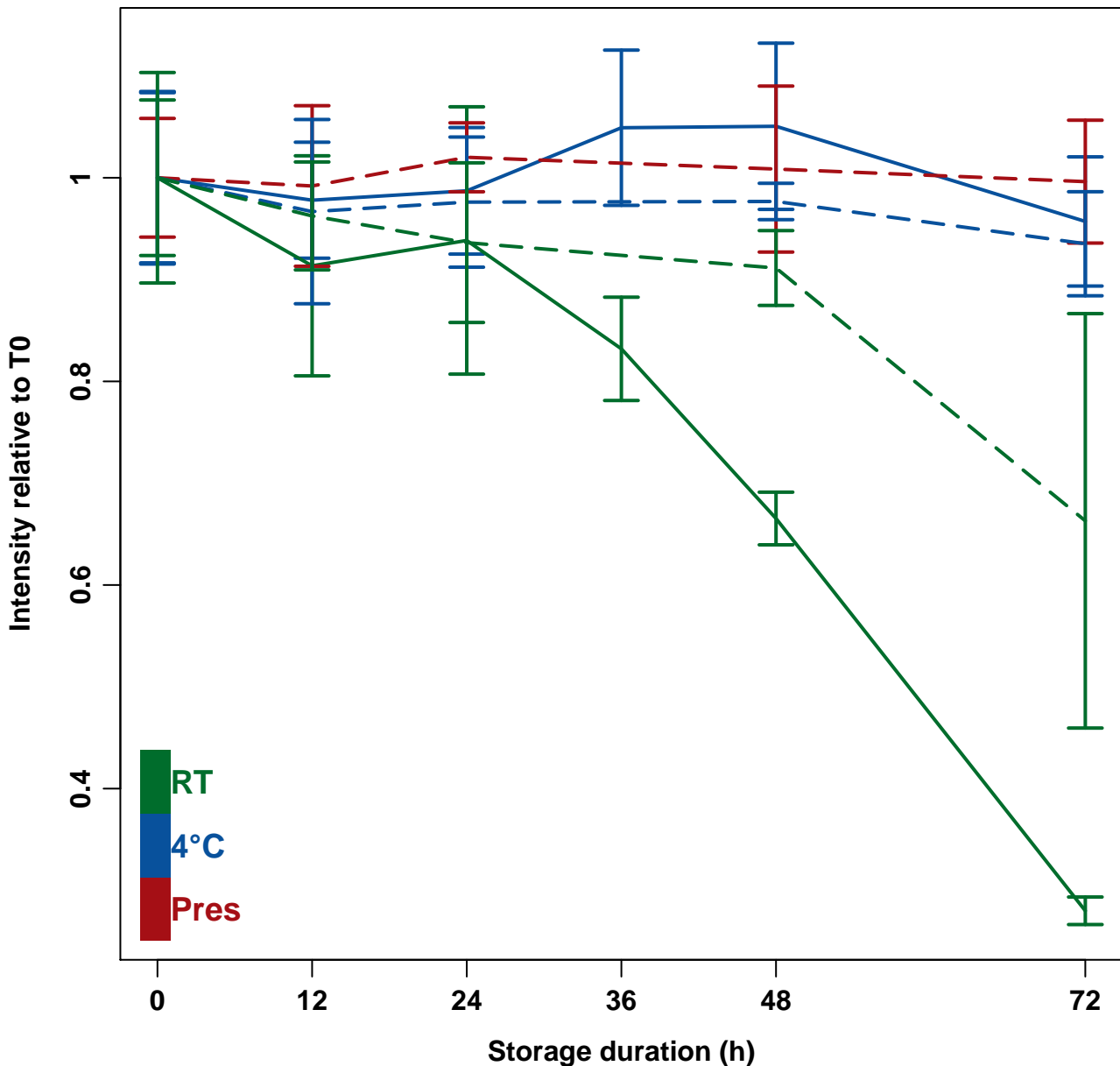

Supplement: Supplementary file 6 — Supplementary material 6 (ZIP 97 kb) [file 11306_2014_764_MOESM6_ESM.zip › Dimethylguanosine.pdf]

## Glutamine

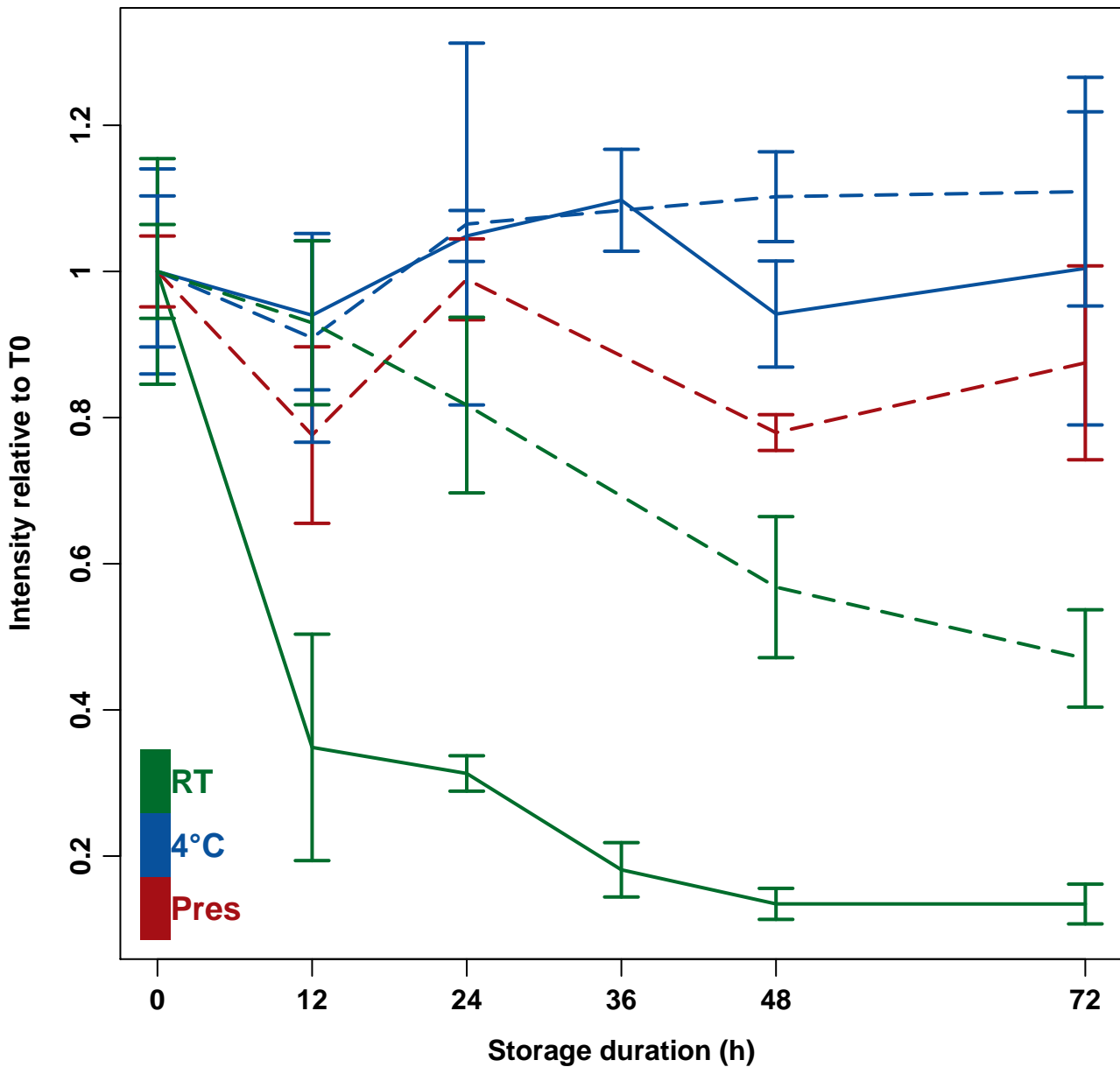

Supplement: Supplementary file 6 — Supplementary material 6 (ZIP 97 kb) [file 11306_2014_764_MOESM6_ESM.zip › Glutamine.pdf]

# Hydroxyretinoic acid glucuronide isomer

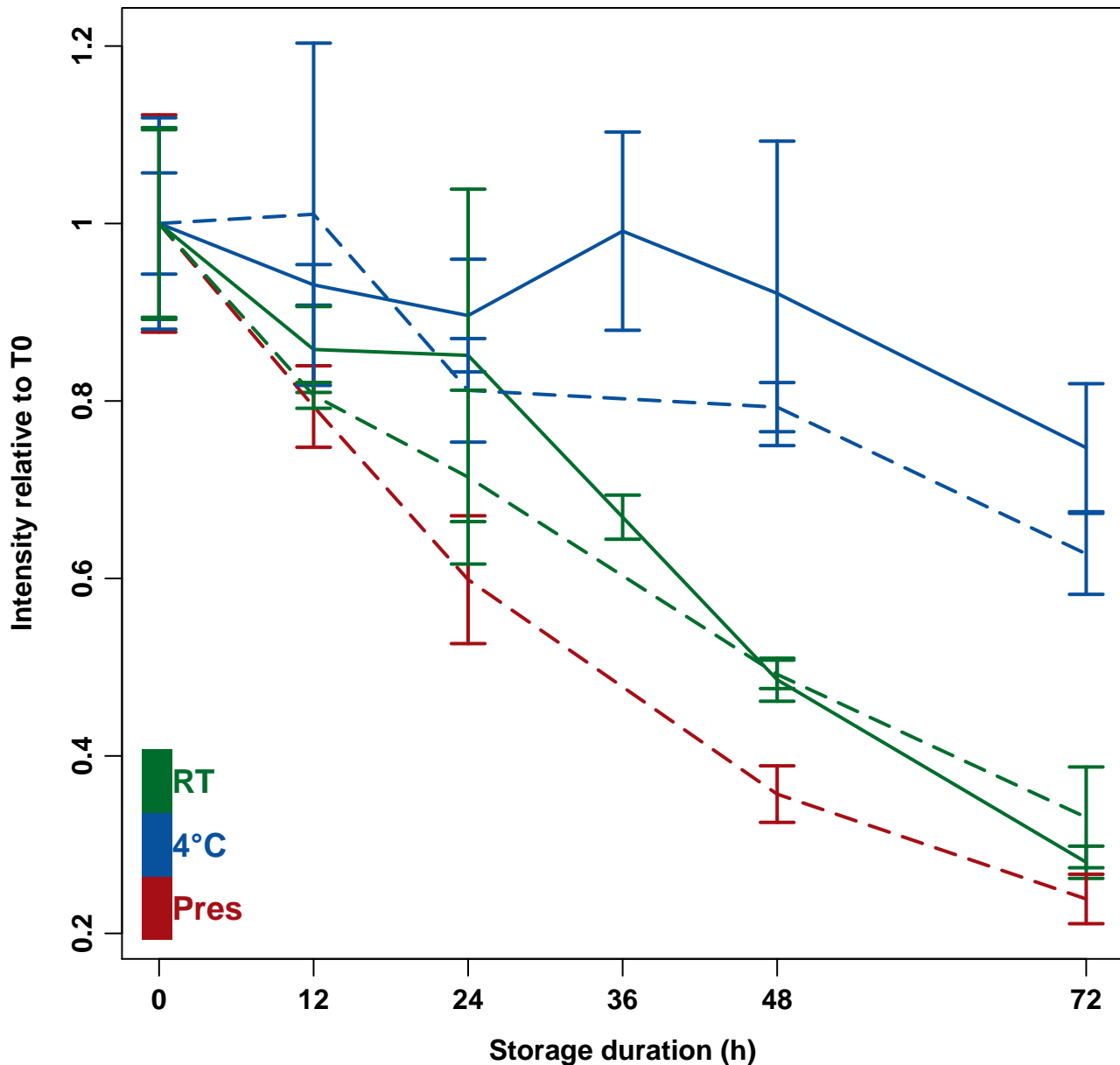

Supplement: Supplementary file 6 — Supplementary material 6 (ZIP 97 kb) [file 11306_2014_764_MOESM6_ESM.zip › Hydroxyretinoic_acid_glucuronide_isomer.pdf]

# Ketoretinoic acid glucuronide isomer

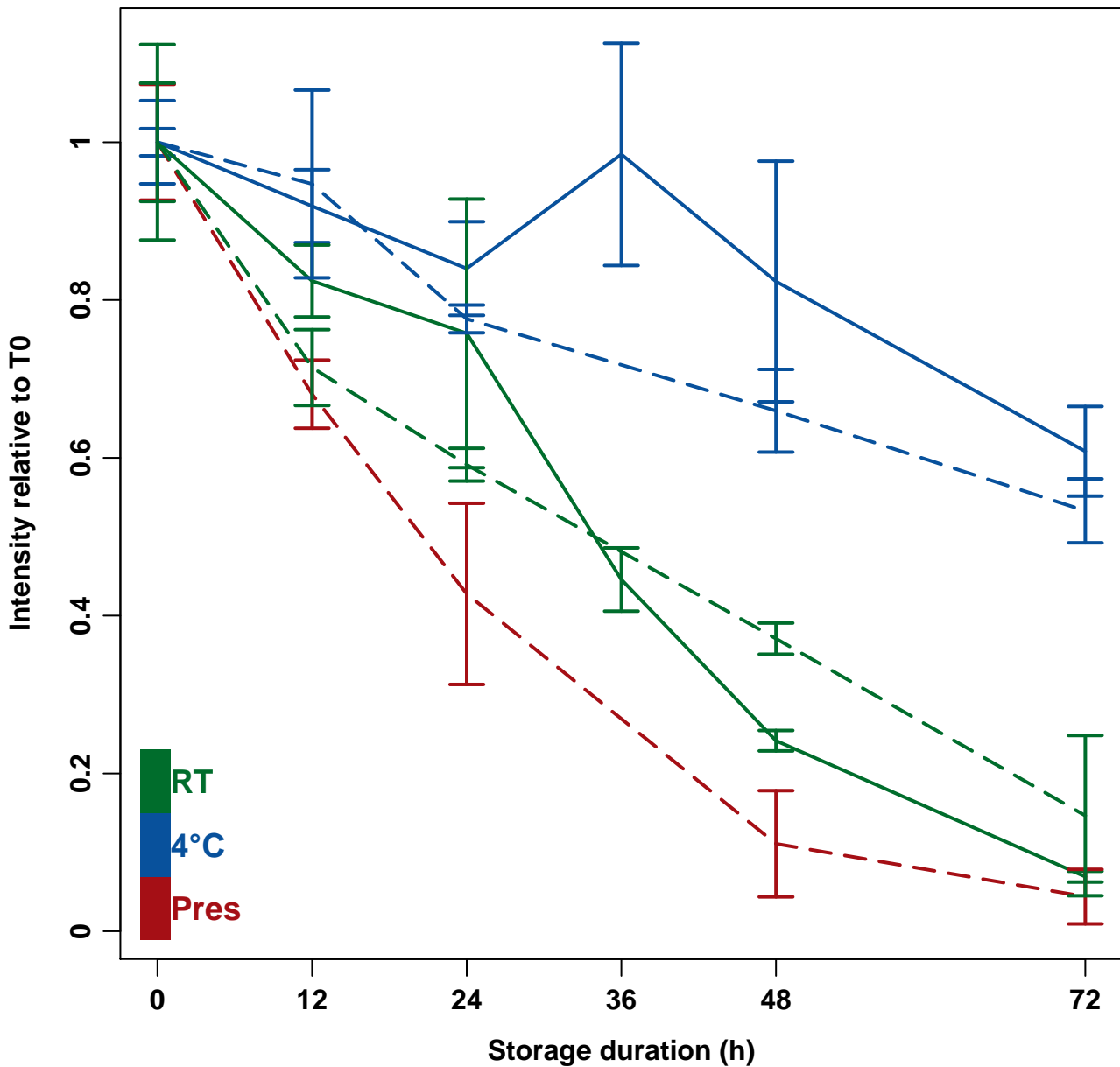

Supplement: Supplementary file 6 — Supplementary material 6 (ZIP 97 kb) [file 11306_2014_764_MOESM6_ESM.zip › Ketoretinoic_acid_glucuronide_isomer.pdf]

# Methylguanosine

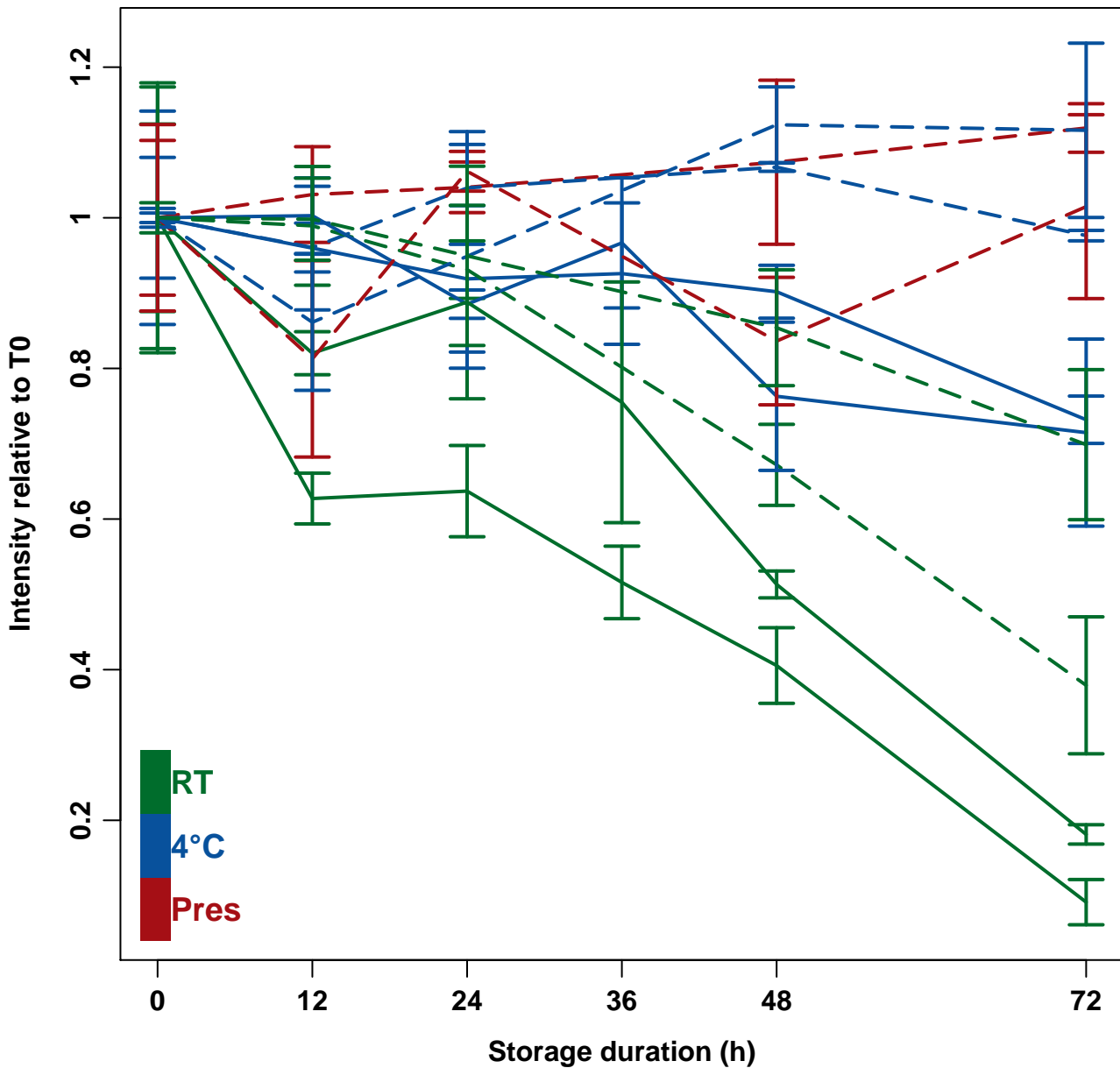

Supplement: Supplementary file 6 — Supplementary material 6 (ZIP 97 kb) [file 11306_2014_764_MOESM6_ESM.zip › Methylguanosine.pdf]

# Methylinosine

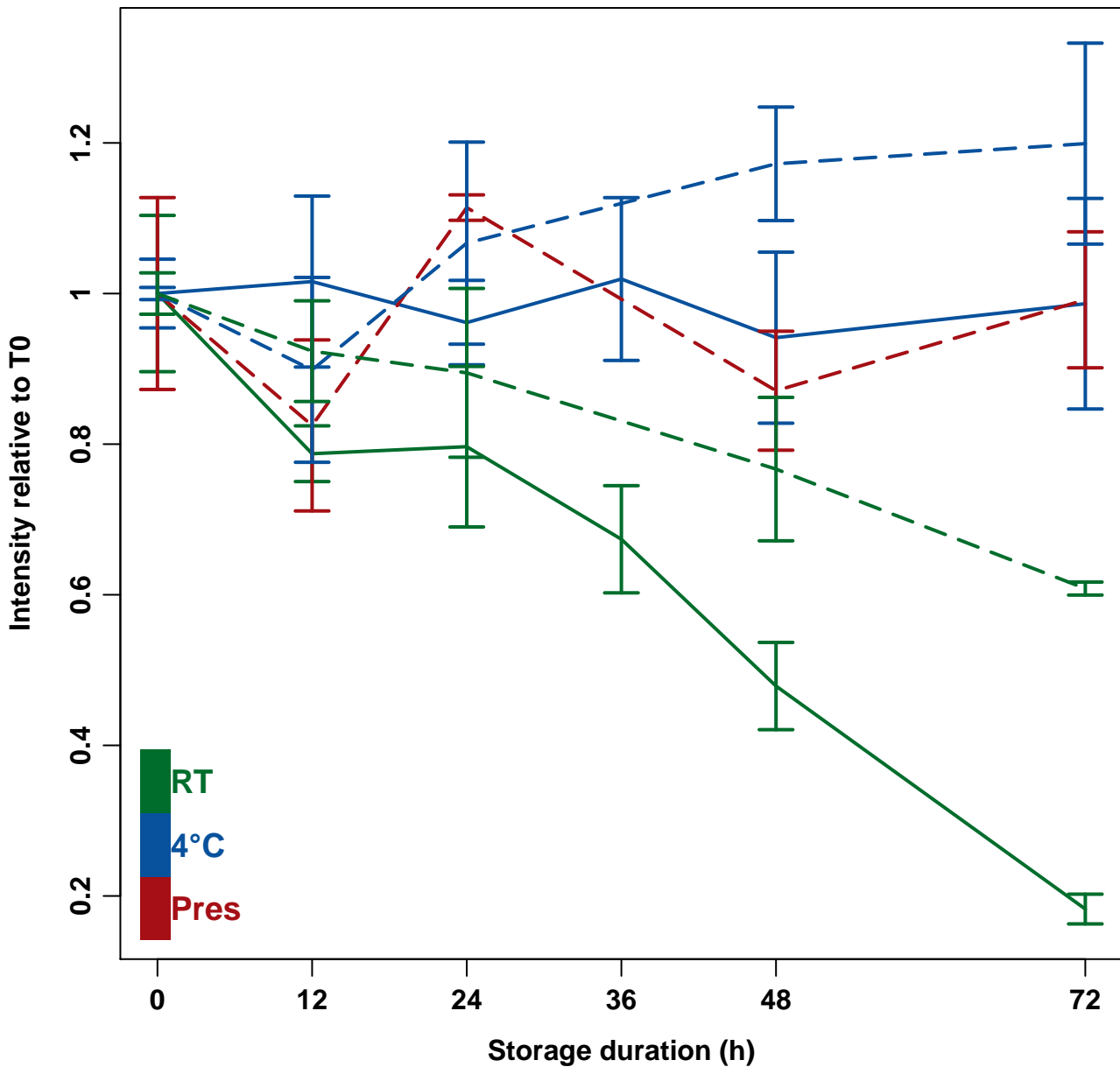

Supplement: Supplementary file 6 — Supplementary material 6 (ZIP 97 kb) [file 11306_2014_764_MOESM6_ESM.zip › Methylinosine.pdf]

# N-Acetylcytidine

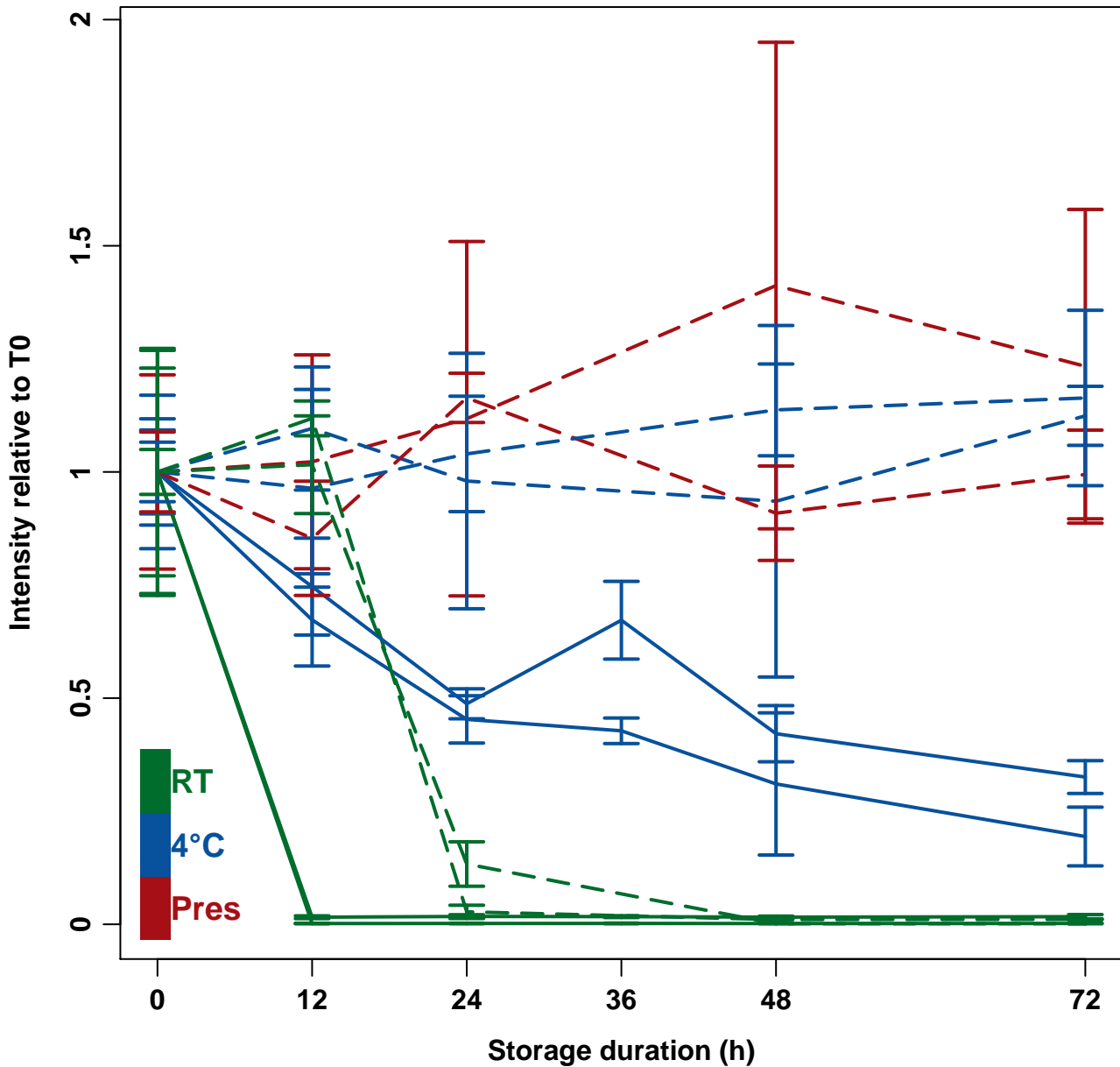

Supplement: Supplementary file 6 — Supplementary material 6 (ZIP 97 kb) [file 11306_2014_764_MOESM6_ESM.zip › N-Acetylcytidine.pdf]

# Orotic acid

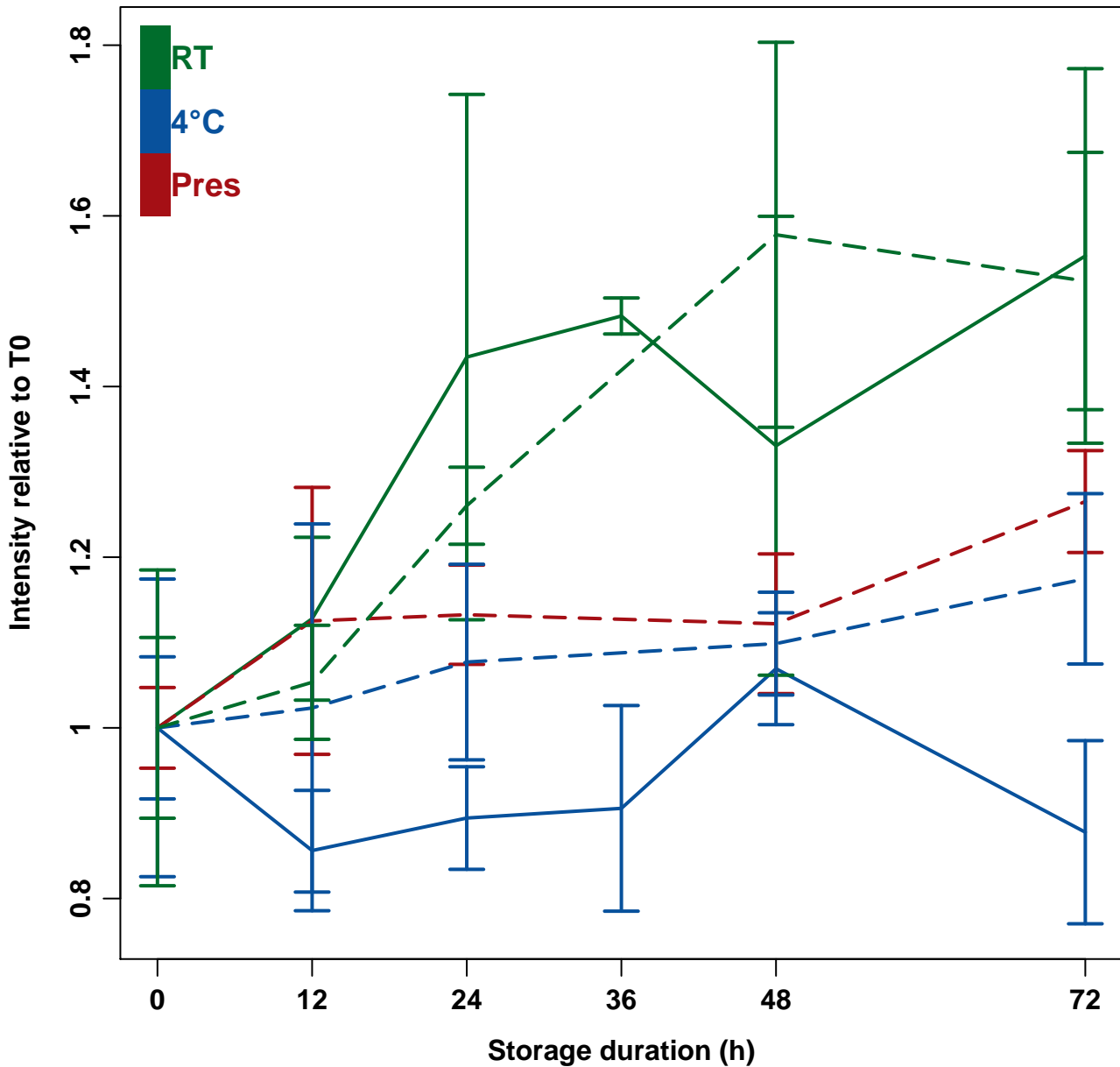

Supplement: Supplementary file 6 — Supplementary material 6 (ZIP 97 kb) [file 11306_2014_764_MOESM6_ESM.zip › Orotic_acid.pdf]

# Threonolactone

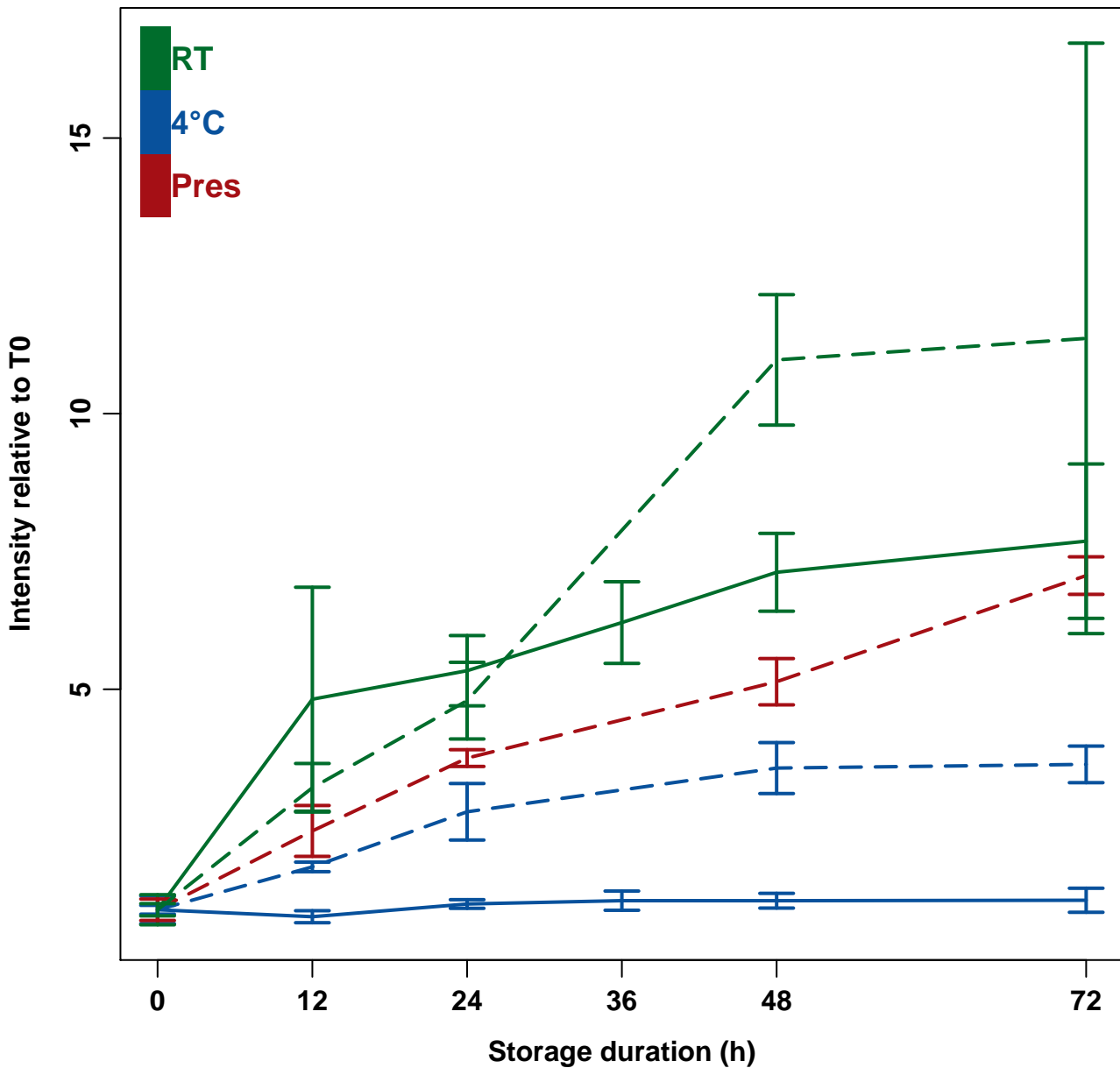

Supplement: Supplementary file 6 — Supplementary material 6 (ZIP 97 kb) [file 11306_2014_764_MOESM6_ESM.zip › Threonolactone.pdf]

# Trimethylamine oxide

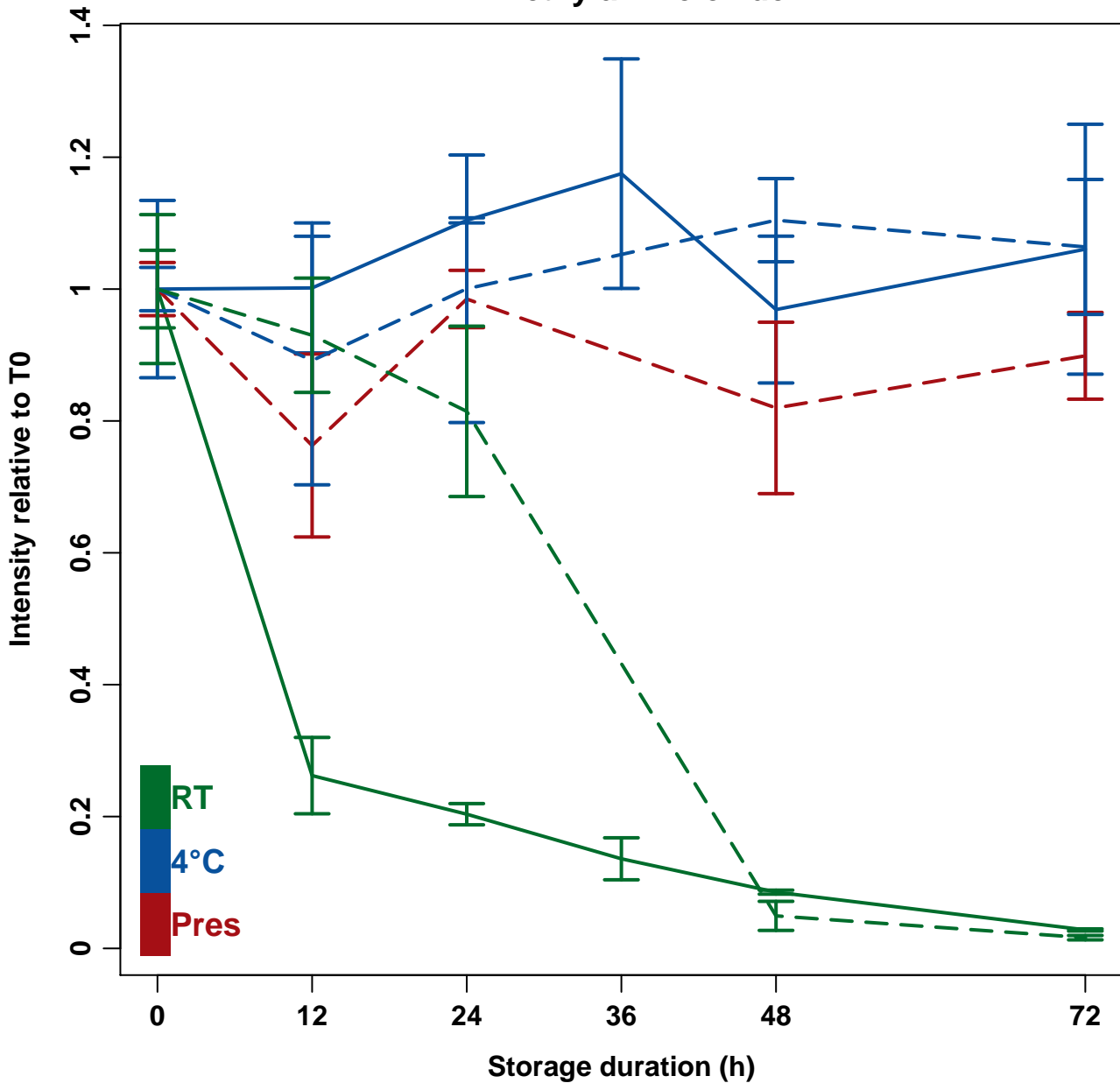

Supplement: Supplementary file 6 — Supplementary material 6 (ZIP 97 kb) [file 11306_2014_764_MOESM6_ESM.zip › Trimethylamine_oxide.pdf]

# Urobilin

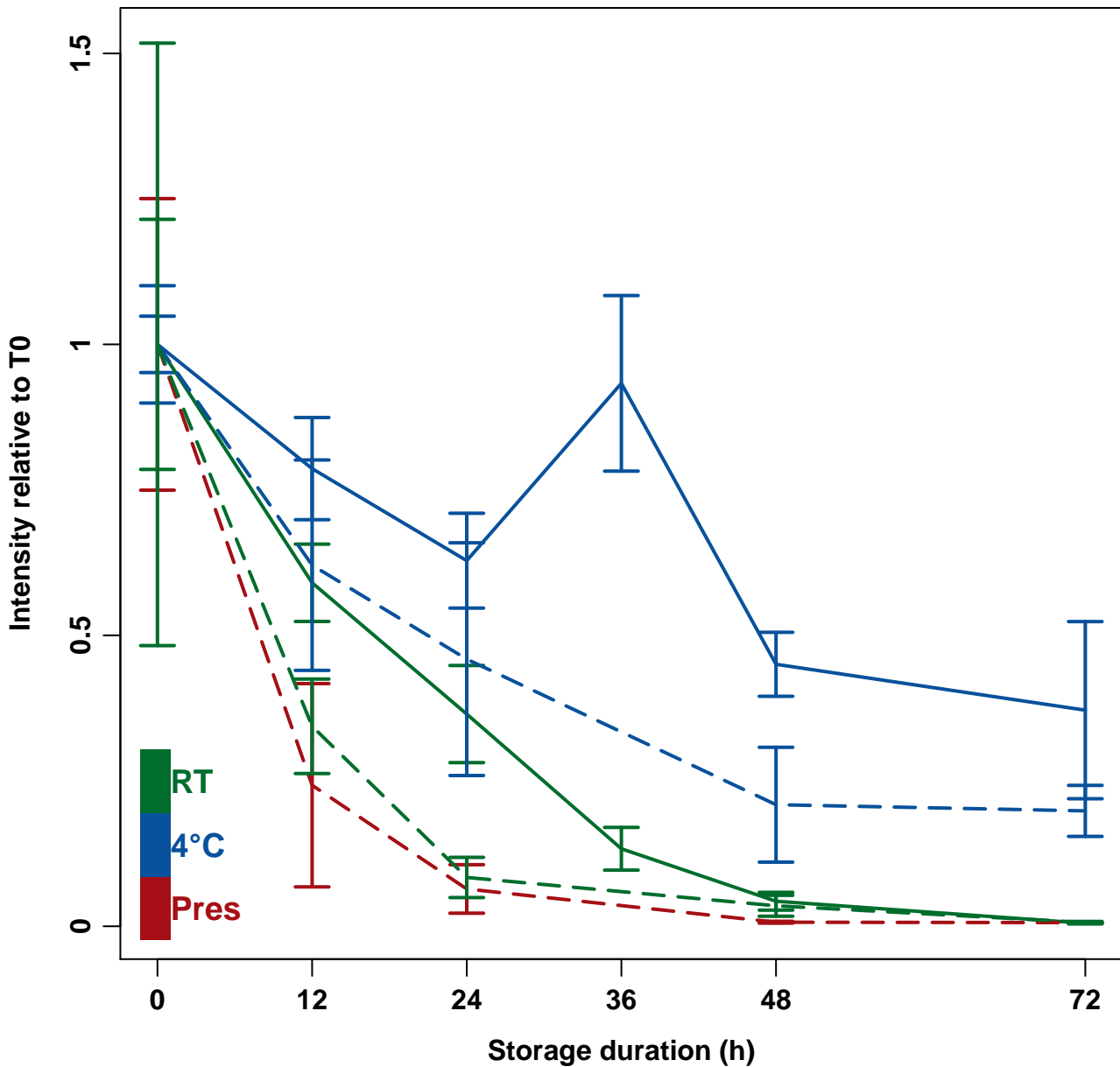

Supplement: Supplementary file 6 — Supplementary material 6 (ZIP 97 kb) [file 11306_2014_764_MOESM6_ESM.zip › Urobilin.pdf]

# Urobilinogen

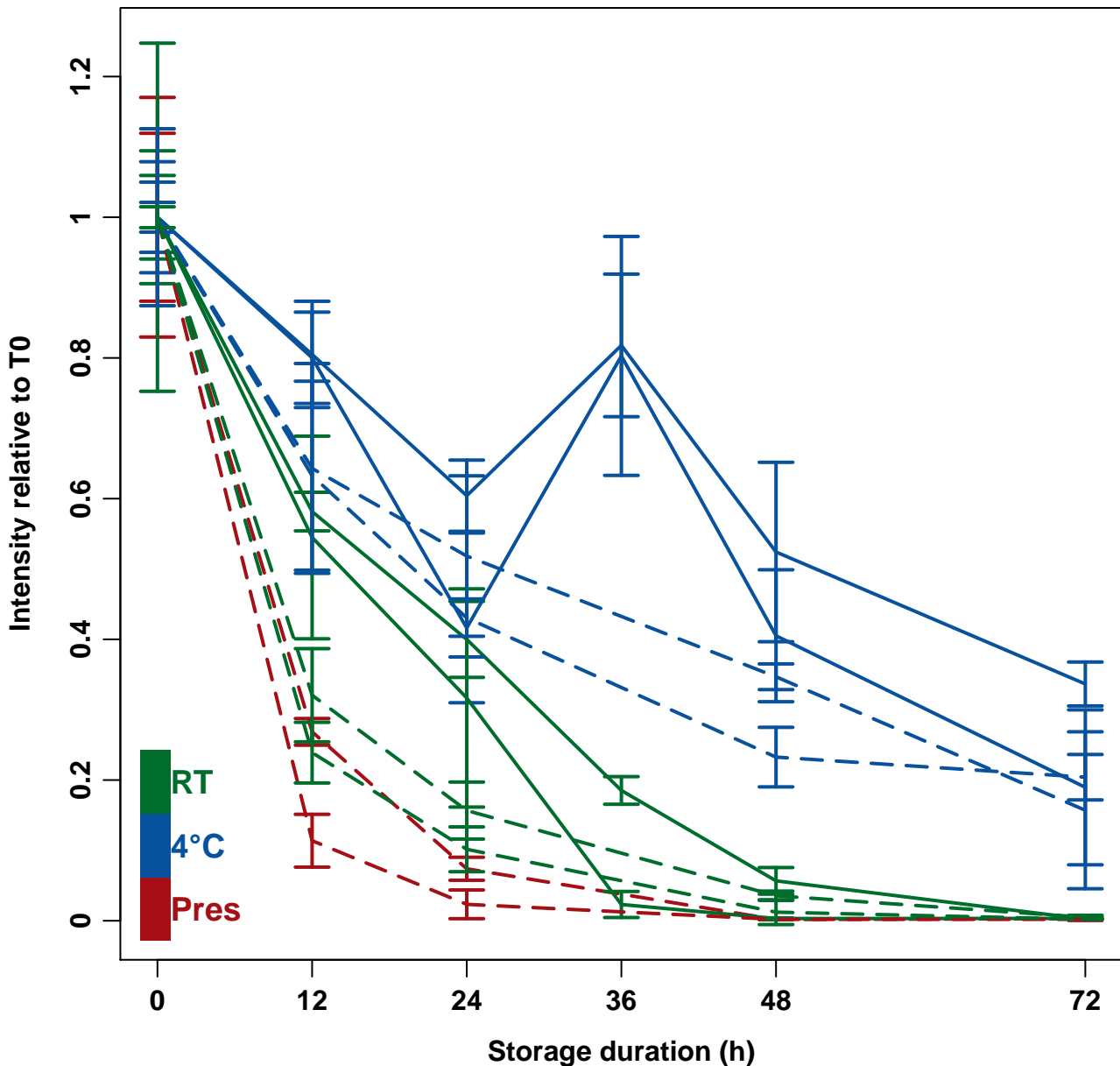

Supplement: Supplementary file 6 — Supplementary material 6 (ZIP 97 kb) [file 11306_2014_764_MOESM6_ESM.zip › Urobilinogen.pdf]

# Valine

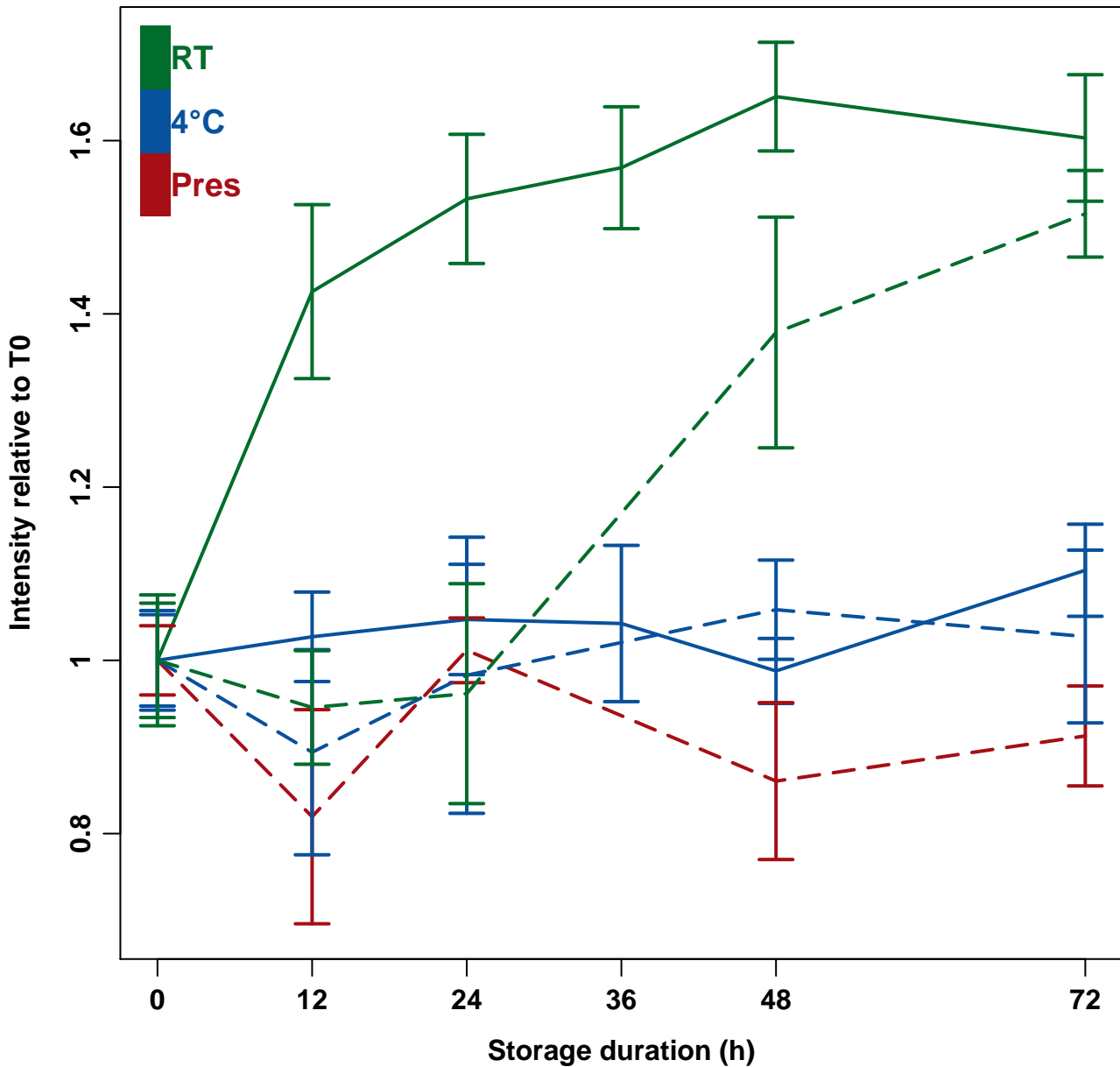

Supplement: Supplementary file 6 — Supplementary material 6 (ZIP 97 kb) [file 11306_2014_764_MOESM6_ESM.zip › Valine.pdf]

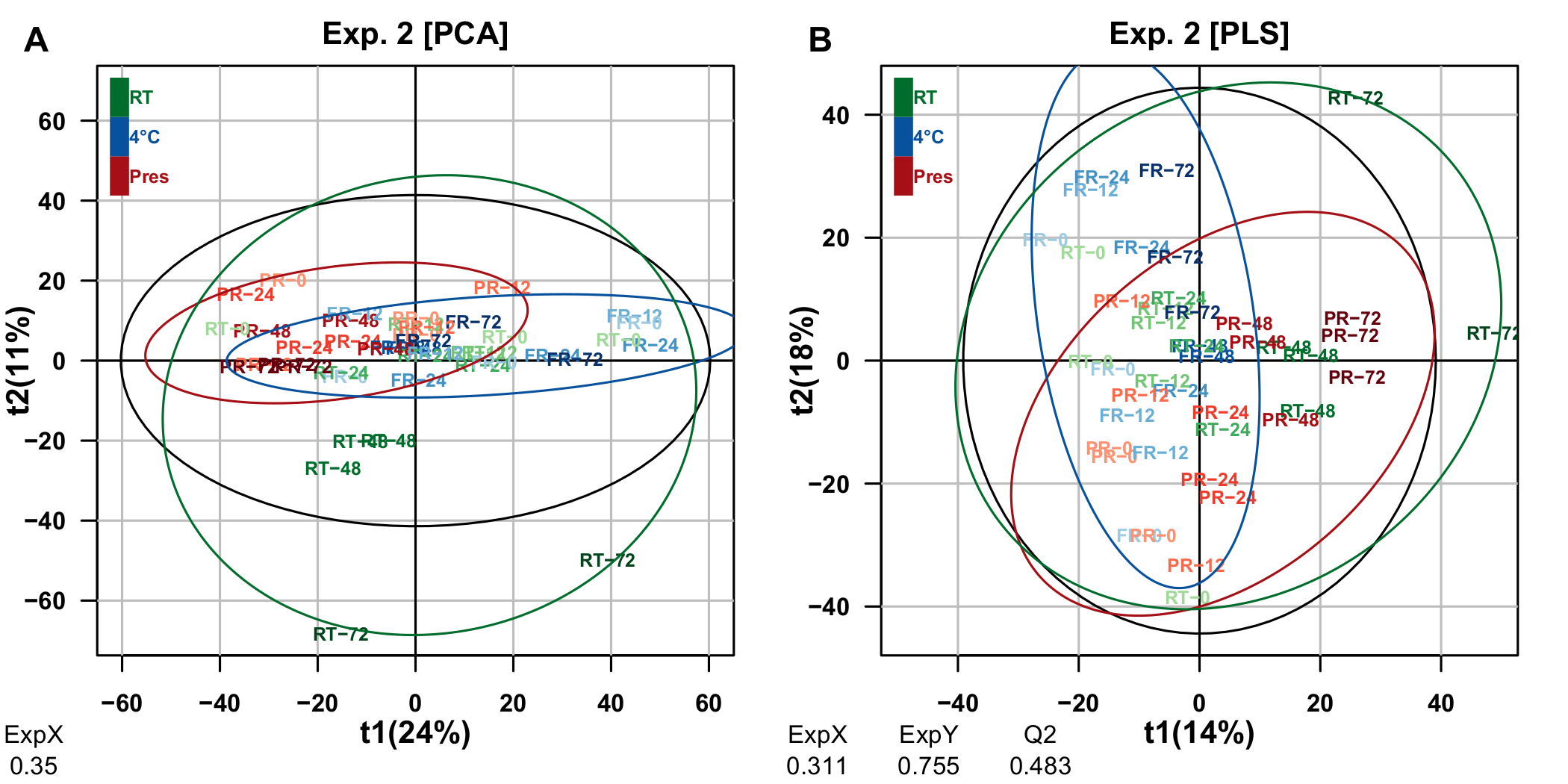

Supplement: Supplementary file 7 — Supplementary material 7 (TIFF 8613 kb) [file 11306_2014_764_MOESM7_ESM.tif]

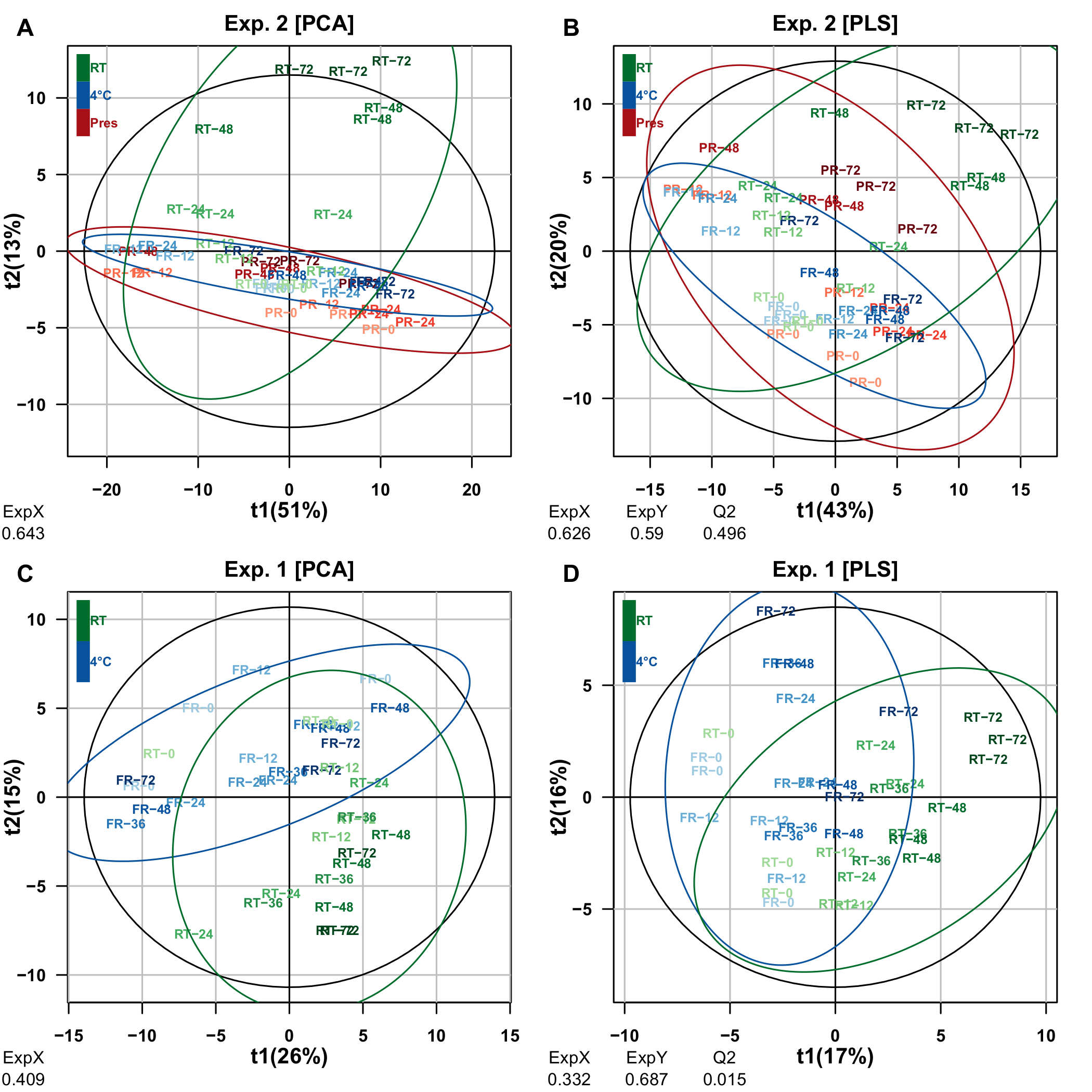

Supplement: Supplementary file 8 — Supplementary material 8 (TIFF 17227 kb) [file 11306_2014_764_MOESM8_ESM.tif]

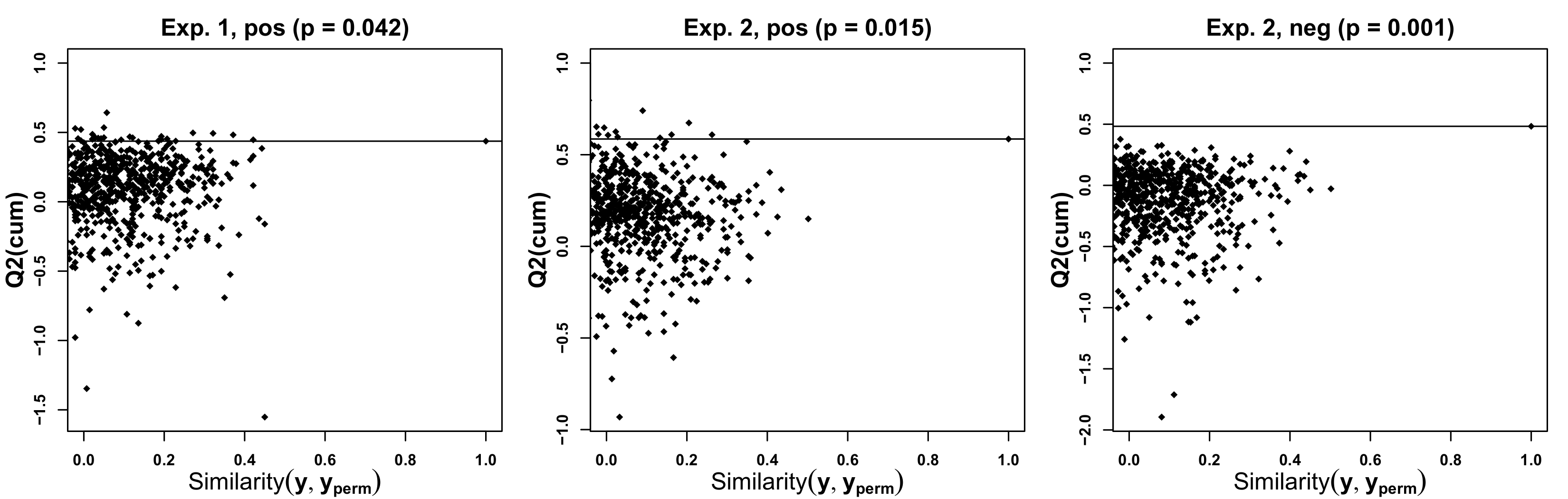

Supplement: Supplementary file 9 — Supplementary material 9 (TIFF 13535 kb) [file 11306_2014_764_MOESM9_ESM.tif]
